# Maternal vitamin B12 deficiency in pregnancy: burden, associated outcomes, and implications for antenatal care in South Asia—a narrative review

**DOI:** 10.3389/fnut.2026.1896162

**Published:** 2026-10-22

**Authors:** Archna Yadav, Kena Yatin Gandhi, Manju Puri

**Affiliations:** 1 Department of Anthropology, University of Delhi, New Delhi, India; 2 Department of Obstetrics and Gynaecology, Gujarat Medical Education and Research Medical College and Hospital Dharpur, Patan, India; 3 Department of Obstetrics and Gynaecology, SGT University Faculty of Medicine and Health Sciences, Gurugram, India

**Keywords:** antenatal care, cobalamin, holotranscobalamin, MATCOBIND, one-carbon metabolism, pregnancy outcomes, South Asia, supplementation

## Abstract

Vitamin B12 (cobalamin) deficiency during pregnancy carries a disproportionate burden in South Asia, yet it remains outside India's national antenatal supplementation programme, which continues to prioritize iron and folic acid alone. This narrative review is organized around three questions of programmatic relevance: how large is the burden of maternal vitamin B12 deficiency and what drives it; what maternal and offspring outcomes are associated with low maternal vitamin B12 status, and how strong is the supporting evidence; and what these findings imply for antenatal practice and policy in resource-limited South Asian settings. Following a documented literature search of two databases (January 2000–March 2026), we synthesize evidence on prevalence, dietary and socioeconomic determinants, biological mechanisms, and clinical outcomes, and appraise the strength of inference underpinning each major claim. Pooled global prevalence estimates range from approximately 17% to 25%, whereas Indian and South Asian estimates in pregnancy commonly reach 40–70%. Low maternal vitamin B12 status has been associated predominantly through observational evidence with preeclampsia, pregnancy loss, gestational diabetes, neural tube defects, fetal growth restriction, preterm birth, and impaired offspring neurodevelopment, with hyperhomocysteinaemia, disrupted one-carbon metabolism, and altered methylation as the principal proposed pathways. India's folate-only supplementation policy may contribute to a low-vitamin B12/high-folate imbalance that has been associated with adverse metabolic outcomes in offspring. Randomized evidence, including the recent MATCOBIND trial, consistently demonstrates improvement in biochemical markers of vitamin B12 status; however, evidence for corresponding clinical benefits remains limited. On current evidence, a targeted, risk-based approach to screening and supplementation, rather than universal supplementation, is best supported and can be readily integrated into existing antenatal infrastructure. Priorities for future research include pregnancy-specific diagnostic thresholds, adequately powered trials with long-term follow-up, independent replication of recent intervention findings, and cost-effectiveness analyses in South Asian populations.

## 1 Introduction

Vitamin B12 (cobalamin) is an essential cofactor for two reactions of relevance to this review: the remethylation of homocysteine to methionine by methionine synthase, which places vitamin B12 within one-carbon metabolism and the supply of methyl groups, and the conversion of methylmalonyl-CoA to succinyl-CoA, which underpins the use of methylmalonic acid (MMA) as a functional marker of deficiency. These pathways are summarized in Figure 1 and are not described further, as the biochemistry is well established and is not the subject of this synthesis (1–3). Pregnancy further increases the physiological demand for vitamin B12. Expanding maternal erythropoiesis, rapid fetal tissue growth, and active placental transfer all increase cobalamin requirements at a time when circulating maternal concentrations decline by approximately 30% by delivery because of haemodilution and preferential fetal transfer. Consequently, reference ranges derived from non-pregnant adults may underestimate true deficiency during pregnancy (4–6).

**Figure 1:**
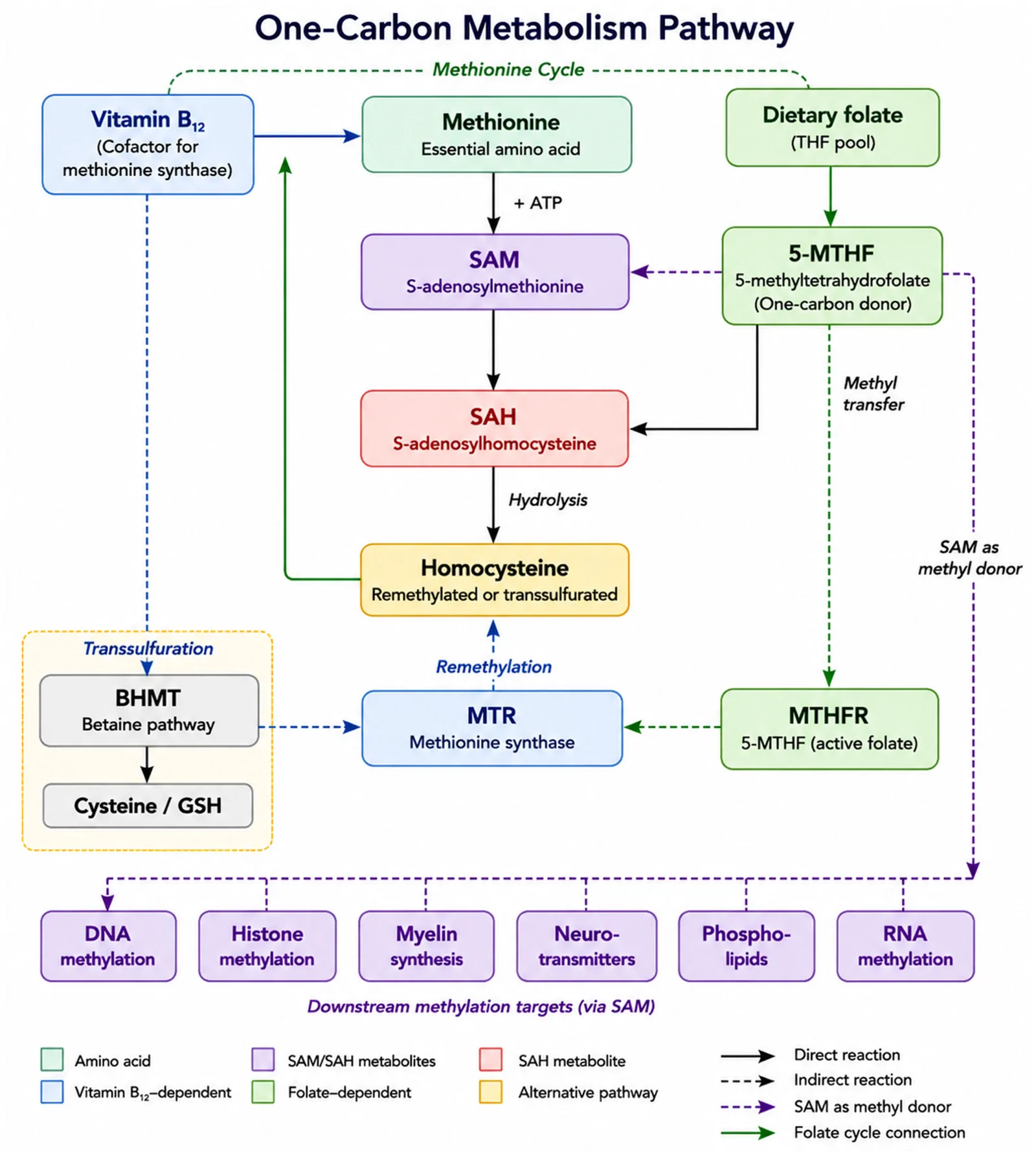
One-carbon metabolism pathway illustrating the interaction between vitamin B12 and folate in homocysteine remethylation and downstream methylation. B12 acts as cofactor for methionine synthase, converting homocysteine to methionine using 5-methyltetrahydrofolate as methyl donor and regenerating tetrahydrofolate for nucleotide synthesis; methionine supplies S-adenosylmethionine for DNA, histone, RNA, and myelin methylation. SAM, S-adenosylmethionine; SAH, S-adenosylhomocysteine; MTHFR, methylenetetrahydrofolate reductase; THF, tetrahydrofolate.

The burden of maternal vitamin B12 deficiency is greatest in South Asia, where animal-source foods, the principal dietary source of cobalamin, are inaccessible to a large proportion of the population because of vegetarian dietary practices and economic barriers. This burden is further compounded by low dietary diversity and recurrent enteric infections (6, 7). Despite prevalence estimates in Indian pregnant women that commonly reach 40–70%, vitamin B12 is absent from India's national antenatal supplementation schedule, which provides iron and folic acid but not cobalamin (6, 8). A further concern is that routine high-dose folic acid can partially correct the anemia of vitamin B12 deficiency while allowing neurological injury to progress, and that a low-vitamin B12/high-folate biochemical state has been associated with adverse metabolic outcomes in offspring (9, 10).

Against this background, the present review is organized around three programmatically relevant questions rather than providing a comprehensive account of cobalamin biology. First, what is the magnitude of the burden of maternal vitamin B12 deficiency, and which factors sustain it? Second, which maternal and offspring outcomes are associated with low maternal vitamin B12 status, through what mechanisms, and critically, how strong is the underlying evidence? Third, what do these findings imply for antenatal screening, supplementation, and policy in South Asia, where the burden is concentrated and resources are constrained? Diagnosis and treatment are discussed only to the extent necessary to address the third question. Throughout, statements of association are kept distinct from causal claims, and the strength of inference behind each major finding is made explicit in the manuscript, allowing readers to interpret the evidence in the context of its quality rather than assigning a uniform level of confidence across a heterogeneous body of literature.

## 2 Methods

### 2.1 Design and reporting

This narrative review synthesizes the available evidence on maternal vitamin B_12_ status during pregnancy, with particular emphasis on its prevalence, assessment, supplementation, and associations with maternal, fetal, neonatal, and offspring outcomes. A narrative review with thematic synthesis was considered more appropriate than a systematic review or meta-analysis because the available literature comprises heterogeneous study designs, including observational studies, randomized controlled trials, mechanistic investigations, and evidence syntheses, with considerable variation in study populations, outcome definitions, and diagnostic thresholds for vitamin B_12_ deficiency. Rather than estimating pooled effect sizes, the objective of this review was to provide a comprehensive overview of the current evidence, identify areas of consistency and uncertainty, and highlight implications for clinical practice and future research. To improve transparency and reproducibility, the search strategy, eligibility criteria, study selection process, and approach to evidence synthesis are described below.

### 2.2 Search strategy

A structured literature search was conducted in PubMed/MEDLINE and Scopus on 20 April 2026 to identify relevant studies published between 1 January 2000 and 31 March 2026. The search strategy comprised three concept blocks combined using the Boolean operator AND: (i) vitamin B_12_ and related one-carbon metabolism biomarkers; (ii) pregnancy, the perinatal period, and lactation; and (iii) maternal, fetal, neonatal, and offspring outcomes, including diagnosis and supplementation. Synonymous terms within each concept block were combined using the Boolean operator OR. Search strategies were adapted to the indexing vocabulary and syntax of each database. The complete database-specific search strategies, applied limits, search dates, and numbers of records retrieved are provided in Supplementary Table S1. Reference lists of relevant systematic reviews, meta-analyses, and key publications were also manually screened to identify additional eligible studies.

Because the primary aim of this review was to characterize the burden of maternal vitamin B_12_ deficiency and its implications for antenatal care in South Asia, particular attention was given to evidence from South Asian countries. The search was not restricted geographically, however, because evidence on maternal and offspring outcomes, diagnostic assessment, biological mechanisms, and supplementation was limited and heterogeneous in South Asian populations. International studies were therefore retained when they provided directly relevant evidence that complemented or contextualized findings from South Asian populations. South Asian relevance was subsequently considered during study selection and thematic synthesis

The search period was restricted to January 2000 onwards to capture contemporary pregnancy research using functional biomarkers of vitamin B_12_ status, including holotranscobalamin, methylmalonic acid, and homocysteine, while retaining more than two decades of relevant evidence.

### 2.3 Eligibility criteria

Studies were considered eligible if they investigated maternal vitamin B_12_ status during pregnancy, its assessment, supplementation, or its relationship with maternal, fetal, neonatal, or offspring outcomes. Eligible publications included original research articles, randomized controlled trials, cohort studies, case-control studies, cross-sectional studies, systematic reviews, meta-analyses, and narrative reviews.

Only studies conducted in human populations and published in English were included. Where relevant to the study objectives, preference was given to studies assessing maternal vitamin B_12_ status using recognized biochemical markers, including serum or plasma vitamin B_12_, holotranscobalamin, methylmalonic acid, and total homocysteine. Conference abstracts lacking sufficient methodological information, editorials, letters, case reports involving fewer than five participants, and studies conducted exclusively in non-pregnant populations without pregnancy-specific relevance were excluded. Studies investigating inherited disorders of cobalamin metabolism were also excluded unless they provided information directly applicable to maternal vitamin B_12_ physiology during pregnancy.

Geographic relevance was considered during study selection and synthesis. Studies conducted in South Asian countries were considered particularly relevant to the primary objectives of this review, including assessment of the burden, associated maternal and offspring outcomes, and implications for antenatal care and policy. Studies from outside South Asia were also included where they provided relevant evidence on maternal or offspring outcomes, diagnostic assessment, biological mechanisms, or supplementation that complemented the available evidence from South Asian populations. Thus, geographic location was not used as an exclusion criterion; rather, South Asian studies were prioritized when drawing conclusions about regional burden and antenatal-care implications, whereas international studies were used selectively to address evidence gaps and provide complementary evidence.

The review aimed to synthesize evidence across epidemiological, clinical, diagnostic, mechanistic, and interventional domains. Consequently, studies were selected based on their relevance to the review objectives, methodological rigor, and contribution to the current understanding of maternal vitamin B_12_ status during pregnancy rather than solely on study design.

### 2.4 Study selection

Records identified through PubMed/MEDLINE and Scopus were imported into EndNote X20 (Clarivate Analytics, Philadelphia, PA, USA), and duplicate records were removed before title and abstract screening. The remaining records were screened against the predefined eligibility criteria, followed by full-text assessment of potentially relevant publications. Study selection was conducted collaboratively by the author team, with records distributed among the authors for initial screening. Borderline or uncertain inclusion/exclusion decisions were reviewed jointly by the authors and resolved through discussion and consensus.

A total of 7,333 records were identified through database searching (PubMed/MEDLINE, *n* = 2,115; Scopus, *n* = 5,218). Following removal of 1,830 duplicate records, 5,503 unique records remained for title and abstract screening. Of these, 4,539 records were excluded because they were not relevant to maternal vitamin B_12_ status or pregnancy-related outcomes. Additional exclusions comprised 80 animal or *in vitro* studies, 39 studies investigating inherited disorders of cobalamin metabolism, 12 case reports involving fewer than five participants, 2 non-English publications, and 2 studies published outside the predefined search period, leaving 829 articles for full-text assessment. The study selection process is summarized in Figure 2.

**Figure 2:**
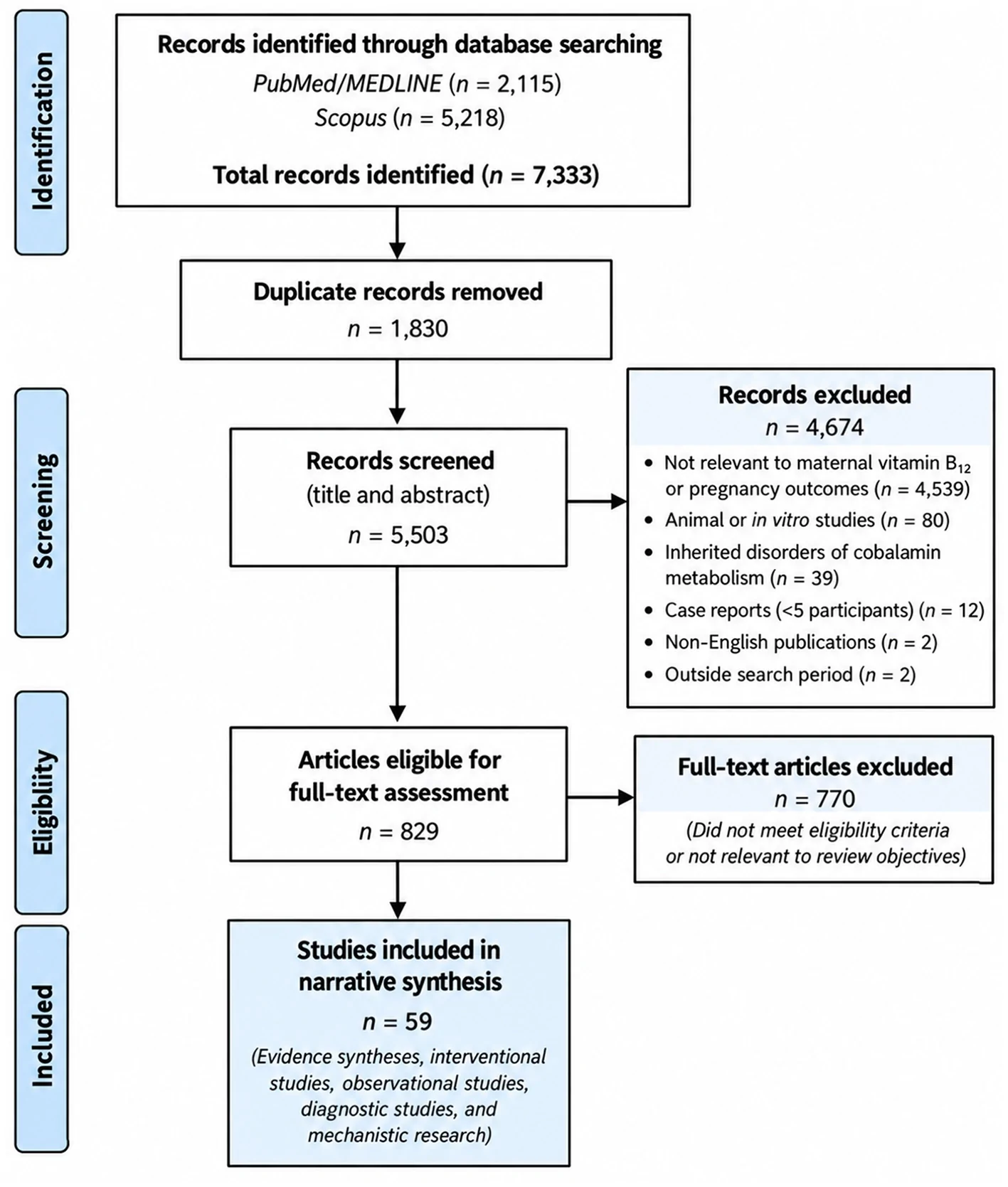
Flow diagram of the literature search and study selection process.

Given the broad scope of this narrative review, the included literature was synthesized according to its relevance to the review objectives. No eligible study was excluded from the narrative synthesis on the basis of study design or evidence level. Rather, “greater interpretative weight” refers to how findings were foregrounded when summarizing the evidence for a particular question. Where a systematic review, meta-analysis, randomized controlled trial, or well-conducted prospective cohort study was available, its findings were considered first when summarizing the overall evidence, while relevant primary studies were retained to provide supporting or contextual evidence. For this purpose, “the same research question” referred to studies addressing the same or closely related exposure-outcome relationship within a comparable clinical context. The designation of evidence as methodologically stronger was based on study design and consistency of findings across studies, rather than on a formal risk-of-bias assessment. Reference lists of eligible reviews and primary studies were also screened manually to identify additional relevant publications.

Ultimately, 59 studies were included in the narrative synthesis. These represented a broad range of study designs, including evidence syntheses, interventional studies, observational investigations, diagnostic studies, and mechanistic research. The characteristics of all included studies, including study design, study population, sample size, principal findings, and references, are summarized in Table 1.

**Table 1:** Characteristics of the studies included in the narrative synthesis (*n* = 59).

Study	Country/Region	Population/Setting	Design	*n*	Study period	Biomarker assessment & cut-offs	Outcome type	Key finding
A. Evidence relevant to South Asia								
A1. Prevalence and burden of maternal vitamin B12 deficiency								
Samuel et al. (96)	Bangalore, Karnataka, South India	Pregnant women; age: ≥18 years; ≤ 14 weeks' gestation; urban South Indian population	Cross-sectional study	366	December 2008–January 2011	Low vitamin B12 < 150 pmol/L; impaired status = low B12 + MMA >0.26 μmol/L	Prevalence/burden	Low plasma vitamin B12 was observed in 51.1%; 42.4% had impaired B12 status
Sukumar et al. (11)	Global	Pregnant women; age: 18–45 years; ethnicity: NR; community and hospital settings	SR/MA	30,762 (57 studies)	NR	Study-specific cut-offs (59–295 pmol/L)	Prevalence/burden	Pooled vitamin B12 insufficiency was approximately 25% (highest rates in Indian subcontinent and Eastern Mediterranean)
Barney et al. (13)	Vellore, Tamil Nadu, South India	Multigravida pregnant women; Mean age: 26 years; ≤ 20 weeks' gestation; rural Kaniyambadi block, Vellore	Community-based cross-sectional study	120	August 2017–May 2018	Serum vitamin B12; deficiency < 200 pg/mL	Prevalence/burden	Vitamin B12 deficiency prevalence was 55%
Behere et al. (6)	India (multi-region)	Pregnant women and offspring; age: NR; ethnicity: NR; rural and urban community- and hospital-based populations	SR (GRADE-assessed)	46 studies (26 cohort, 13 case-control, 7 RCTs)	NR	Study-specific cut-offs; not harmonized	Prevalence/burden	Vitamin B12 deficiency prevalence in Indian pregnant women was 40–70% in 3 studies
Finkelstein et al. (17)	Southern India (Chittoor, Andhra Pradesh)	Women of reproductive age; age: 15–40 years; non-pregnant, non-lactating; rural and urban households	Population-based cross-sectional biomarker survey	979	NR	Vitamin B12 deficiency < 148 pmol/L; insufficiency < 221 pmol/L	Prevalence/burden	Vitamin B12 deficiency was 48.3% and insufficiency 74.3%
Das et al. (16)	Ambala District, Haryana, North India	Women of reproductive age; age: 18–49 years; non-pregnant, non-lactating; rural setting	Population-based cross-sectional survey	775	2017	Serum vitamin B12 deficiency < 200 pg/mL; marginal deficiency 200–300 pg/mL	Prevalence/burden	Vitamin B12 deficiency was 57.7% and marginal deficiency 23.5%
Maji et al. (59)	West Bengal, India	Lactating women; mean age: 25.65 ± 3.71 years; six weeks postpartum; tertiary-care hospital setting	Observational cross-sectional study	121	July–August 2022	Serum vitamin B12 deficiency < 200 pg/mL (150 pmol/L)	Prevalence/burden	Vitamin B12 deficiency was 15.7%
Nguyen et al. (12)	Global (43 studies; 28 countries)	Pregnant women; age: NR; ethnicity: NR; multiple geographic populations	SR/MA of observational studies	72,635 (43 studies)	NR	Study-specific vitamin B12 deficiency cut-offs	Prevalence/burden	Pooled vitamin B12 deficiency prevalence was 17.1% (95% CI 8.8–30.6%).
A2. Maternal pregnancy outcomes								
Puri et al. (30)	North India (New Delhi)	Women with RPL; age: 18–36 years; ethnicity: North Indian	Case-control study	450 participants	October 2006–August 2010	Serum vitamin B12 measured by chemiluminescence; deficiency defined as < 200 pg/mL	Pregnancy loss/RPL	B12 deficiency and hyperhomocysteinemia were significant risk factors for RPL (OR 16.39 and 7.02, respectively); MTHFR C677T was associated with increased homocysteine among cases.
Kaur et al. (31)	North India (New Delhi)	Non-pregnant women with RPL; 105 cases and 135 multiparous controls; age: NR; ethnicity: North Indian	Case-control study	240 participants	NR	Serum vitamin B12; deficiency defined as < 200 pg/mL	Pregnancy loss/RPL	B12 deficiency, folate/B12 imbalance and altered homocysteine status were observed in women with RPL.
Mishra et al. (32)	North India (New Delhi)	Pregnant women; age: 19–36 years; recruited at 7–13 weeks' gestation and followed through pregnancy to outcome; ethnicity: North Indian	Prospective follow-up cohort study	100 participants	NR	Serum vitamin B12; deficiency defined as < 220 pg/mL	Pregnancy outcomes/adverse pregnancy outcomes, including LBW	First-/second-trimester hyperhomocysteinemia was associated with >3-fold higher risk of adverse pregnancy outcomes; B12 deficiency was associated with LBW.
Bala et al. (29)	Varanasi, Uttar Pradesh, India	Women with early pregnancy loss and healthy pregnant controls; age: NR; ethnicity: NR	Case-control study + MA	153 participants (83 cases, 70 controls)	NR	Plasma vitamin B12; deficiency/low-B12 criterion: NR	Pregnancy loss/early pregnancy loss	Low B12 and hyperhomocysteinemia were independently associated with early pregnancy loss; their combination was associated with higher risk (OR 4.98, *P* = 0.002). Folate deficiency was not associated with early pregnancy loss.
Mardali et al. (28)	Global (Asia, Africa, and America)	Pregnant women with preeclampsia and normotensive pregnant women; age: 18–34 years; ethnicity: diverse across included studies	SR/MA of case-control studies	3,211 participants (1,390 preeclampsia cases; 1,821 controls)	NR	Serum vitamin B12 concentration; study-specific methods/cut-offs	Hypertensive disorders/preeclampsia	Maternal serum B12 was significantly lower in women with preeclampsia than in normotensive women (MD −15.24 pg/mL, 95% CI −27.52 to −2.95).
He et al. (33)	Global (China, India, Australia, and Britain)	Pregnant women; age: NR; ethnicity: diverse across included studies	SR/MA of 10 observational studies (4 prospective cohorts, 2 cross-sectional, 4 case-control)	10,595 participants	NR	Serum vitamin B12; study-specific cut-offs, primarily 150–220 pmol/L	Gestational diabetes mellitus	Among prospective cohorts, maternal B12 deficiency was associated with increased GDM risk (OR 1.46, 95% CI 1.21–1.79).
Li N. et al. (35)	Global	Pregnant women; age: NR; ethnicity: diverse across included studies	Dose-response MA of 13 observational studies	26 datasets from 13 studies	NR	Plasma/serum vitamin B12; study-specific assessment methods/cut-offs	Gestational diabetes mellitus	No significant association between maternal plasma/serum B12 and GDM risk (OR 0.77, 95% CI 0.58–1.02; very low-certainty evidence).
Ma et al. (25)	Zhengzhou, Henan, China	Pregnant Chinese women with preeclampsia and hospital-based healthy pregnant controls; ethnicity: NR	Case-control study	880 (440 mother-infant pairs)	March 2016–June 2019	Maternal dietary vitamin B12 intake assessed using a validated 78-item semiquantitative food-frequency questionnaire; no B12 deficiency cut-off	Dietary B12 intake; preeclampsia	Higher dietary B12 intake was associated with lower odds of preeclampsia (highest vs. lowest quartile, adjusted OR 0.68, 95% CI 0.52–0.88).
Sobczynska-Malefora et al. (36)	London, UK (Includes South asian population)	Pregnant British-Bangladeshi women; mean age: 29 years; third trimester (mean gestational age 33 weeks)	Cross-sectional	59 (24 with GDM and 35 with normal glucose tolerance)	NR	Serum total B12, HoloTC, MMA and homocysteine; serum B12 reference range 140–664 pmol/L; B12 deficiency suggested if HoloTC < 25 pmol/L and MMA >280 nmol/L	B12 status; insulin resistance/HOMA-IR and GDM	HOMA-IR was inversely correlated with total B12 and HoloTC; total B12 remained an independent predictor of HOMA-IR after adjustment. Folate markers were associated with insulin resistance.
Chen et al. (34)	Global (Europe and Asia)	Pregnant women; age: NR; ethnicity: diverse across included studies	SR/MA of 15 observational studies	15,190 participants	NR	Maternal vitamin B12 concentration/deficiency; study-specific definitions and assessment periods	Gestational diabetes mellitus	B12 deficiency in the second/third trimester was associated with increased GDM risk (OR 1.59, 95% CI 1.10–2.29); stronger in Asian populations (OR 2.08, 95% CI 1.47–2.96). A higher folate: B12 ratio was also associated with increased GDM risk.
Zhang et al. (26)	Shanghai, China	Pregnant Chinese Han women; age: >18 years; ethnicity: Chinese Han; receiving antenatal care; all supplemented with 800 μg folate + 4 μg vitamin B12 daily	Real-world case-control analysis using hospital maternal database	1,178 (1,006 normotensive, 131 pregnancy-induced hypertension (PIH), 41 preeclampsia)	June 2018–August 2021	Serum B12 measured at 10–14 weeks; reference range 138–652 pmol/L; no specific B12 deficiency cut-off reported	One-carbon metabolic indicators; pregnancy-induced hypertension and preeclampsia	A model incorporating homocysteine, folate and BMI predicted PIH particularly well in women with the MTHFR CC genotype (AUC 0.802); lower folate predicted progression to preeclampsia. B12 showed no association with PIH/preeclampsia.
A3. Fetal growth and birth outcomes								
Muthayya et al. (43)	Bengaluru, Karnataka, India	Pregnant women; age: 17–40 years; recruited before 20 weeks' gestation	Prospective observational cohort	478	Nov 2001–Aug 2003	Serum vitamin B12; analyzed by trimester-specific tertiles	Fetal growth/IUGR	Women in the lowest B12 tertile had higher odds of IUGR across pregnancy, strongest in the second trimester (AOR 9.28).
Dwarkanath et al. (86)	Bengaluru, Karnataka, India	Pregnant women; age: 17–40 years; recruited before 13 weeks' gestation; urban population with diverse socioeconomic backgrounds	Prospective observational cohort	1,838	NR	Dietary B12 and total folate intake assessed by FFQ; B12 inadequacy < 1.2 μg/day; B12:total folate intake ratio examined	Fetal growth/SGA	Low first-trimester B12 and folate intakes were independently associated with higher SGA risk. Among women with high supplemental folic acid intake, a lower vitamin B12: total folate intake ratio was associated with higher SGA risk (adjusted RR 2.73, 95% CI 1.17–6.37); a similar trend was observed in a blood micronutrient subset (*n* = 316).
Sukla et al. (48)	Varanasi, Uttar Pradesh, North India	Mother–newborn pairs; cord-blood/plasma samples; age/ethnicity: NR	Prospective cohort	334 for B12/folate biochemical analysis (total cohort = 421)	Jan 2010–Dec 2012	Vitamin B12 measured by chemiluminescence; deficiency cut-off NR	Low birth weight/neonatal hyperbilirubinaemia	B12 deficiency was reported in 65% of newborns; hyperhomocysteinemia and MTHFR 677T were associated with LBW, while MTHFR 677T and folate deficiency predicted neonatal hyperbilirubinaemia.
Rogne et al. (44)	Global (North America, Europe, Africa, Oceania and Asia)	Pregnant women from longitudinal cohort studies; population characteristics varied across included studies	SR + IPD MA	11,993 observations; 11,216 in IPD MA (18 studies; 22 eligible)	NR	Vitamin B12 deficiency < 148 pmol/L	Low birth weight/preterm birth	Maternal B12 deficiency was associated with increased LBW risk (aRR 1.15, 95% CI 1.01–1.31); higher B12 was associated with lower preterm-birth risk.
Behera et al. (45)	Cuttack, Odisha, Eastern India	Pregnant women; age: 17–40 years; attending a tertiary-care hospital	Prospective cohort	180 (90 low B12; 90 normal B12)	Sep 2020–Aug 2021	Serum vitamin B12; < 200 pg/mL deficient; ≥200 pg/mL normal	Fetal growth/birth outcomes	Low maternal B12 status was associated with impaired fetal growth and adverse birth outcomes.
Barney et al. (91)	Rural South India (Kaniyambadi, Tamil Nadu)	Pregnant women; recruited at ≤ 20 weeks' gestation; age/ethnicity: NR	Cross-sectional longitudinal study	120	NR	Vitamin B12 assessment/cut-off NR	Abortion/preterm birth/LBW	Adverse outcomes included abortion (5%), preterm birth (4.2%) and LBW (8.8%); MTHFR c.1262G>T was associated with adverse perinatal outcomes.
Deepa et al. (46)	Bengaluru, urban India (MAASTHI cohort)	Pregnant women; age: 18–45 years; recruited from public hospitals; samples collected at 24–32 weeks' gestation	Prospective birth cohort	230	2016–2019	Serum vitamin B12 by CMIA; >221 pmol/L adequate, 148–221 pmol/L low, < 148 pmol/L deficient	Birth weight/preterm delivery	Maternal B12 status was not significantly associated with LBW or preterm delivery; vitamin B6 and impaired folate status were associated with LBW.
Gerety et al. (50)	Rural Bangladesh	Pregnant women in a rural prospective cohort; age/ethnicity: NR	Prospective cohort	376	NR	Maternal vitamin B12 status; study-specific definition of insufficiency	Preterm birth	First-trimester B12 insufficiency was independently associated with preterm birth (aOR 3.33).
A4. Congenital anomalies								
Tang et al. (41)	Global (32 studies)	Mothers of NTD-affected pregnancies and controls; Caucasian, Asian and other ethnicities.	MA of 32 case-control studies	5,885 (1,890 NTD-affected mothers; 3,995 controls)	NR	Maternal vitamin B12 levels; measurement methods varied across included studies; no single deficiency cut-off	Neural tube defects	Maternal B12 levels were lower in NTD-affected pregnancies than controls (RoM 0.91, 95% CI 0.87–0.95); folate was also lower and homocysteine higher.
Munger et al. (92)	Tamil Nadu, India	Mothers of children with isolated cleft lip with or without cleft palate	Case-control	97 (47 cases and 50 controls)	NR	Plasma vitamin B12 measured by electrochemiluminescence; primary analysis based on median B12 (low vs high); marginal B12 < 221 pmol/L	Orofacial clefts	Low maternal plasma B12 was associated with CL±P (OR 2.48, 95% CI 1.02–6.01); low B12 with high MMA showed a stronger association (OR 6.54, 95% CI 1.33–32.09).
Jahan et al. (93)	Bangladesh (Dhaka)	Pregnant women; age: 18–40 years; 28–40 weeks' gestation	Cross-sectional	90	Sep 2022–Aug 2023	Serum vitamin B12; < 200 pg/mL low, ≥200 pg/mL normal	Congenital anomalies/fetal outcomes	Low maternal B12 was associated with higher rates of oligohydramnios, fetal hydrocephalus and NTDs; no significant maternal complications were observed.
Nie et al. (40)	Global (14 countries; includes India)	Mothers with NTD-affected pregnancies and controls	SR/MA of 38 case-control studies	6,614 (2,316 NTD cases; 4,298 controls)	NR	Maternal serum/plasma vitamin B12 concentration; measurement methods varied across studies; no single deficiency cut-off	Neural tube defects	Maternal B12 concentrations were lower in NTD-affected pregnancies than controls (SMD −0.23, 95% CI −0.32 to −0.14); association persisted when maternal folate did not differ (SMD −0.19, 95% CI −0.28 to −0.10).
A5. Offspring neurodevelopment and metabolic outcomes								
Yajnik et al. (37) (Pune)	Pune, Maharashtra, India	Pregnant women from six rural villages; age: NR; ethnicity: NR; maternal–offspring cohort with offspring follow-up to 6 years	Prospective cohort	700 participants	1993–2003	Maternal plasma vitamin B12; low B12 defined as < 150 pmol/L	Offspring adiposity/insulin resistance	Low maternal B12 at 18 weeks was associated with higher offspring insulin resistance at 6 years; high folate/low B12 was associated with the greatest insulin resistance.
Srinivasan et al. (54)	South India (Bengaluru, Karnataka)	Pregnant women; age: ≥18 years; ≤ 14 weeks' gestation; Urban Population; Predominantly lower socioeconomic strata	RCT	366 randomized; 178 infants assessed at 9 months	NR	Plasma vitamin B12 measured by electrochemiluminescence; low vitamin B12 < 150 pmol/L	Offspring neurodevelopment	Maternal B12 supplementation (50 μg/day) produced no significant differences in BSID-III subscales at 9 months; higher maternal tHcy was negatively associated with language and motor scores.
Thomas et al. (55)	South India (Bengaluru, Karnataka)	Children born to mothers aged ≥18 years enrolled in a placebo-controlled maternal vitamin B12 supplementation trial; Urban population	RCT, 30-month follow-up	218	NR	Maternal plasma vitamin B12 measured during pregnancy; low vitamin B12 < 150 pmol/L	Offspring neurodevelopment	Maternal B12 supplementation was associated with higher expressive-language scores (β = 0.14, *P* = 0.03); elevated maternal homocysteine was associated with lower neurodevelopmental scores.
D'Souza et al. (51) (PRIYA)	Pune, Maharashtra, India	Adolescents from the PMNS preconception cohort; Rural Population	RCT	266 females; 74 offspring assessed for neurodevelopment	NR	Circulating vitamin B12 measured at baseline, 6–12 months after supplementation, 28 weeks' gestation and in cord blood; severe B12 deficiency < 100 pmol/L was excluded before randomization	Offspring neurodevelopment	Preconceptional B12 supplementation improved offspring neurodevelopment at 2 years.
Jembere and Dewey (52)	Global (studies from Spain, USA, India, Kenya, Singapore, UK, Netherlands, Nepal, Canada and Mexico)	Mother–child pairs; children < 18 years	Scoping review	23,825	NR	Maternal vitamin B12 assessed through blood status, dietary intake, supplementation or deficiency; definitions varied; lowest reported low-B12 threshold < 77 pmol/L	Offspring neurodevelopment (cognitive, language, motor and brain development)	Evidence for an association between prenatal maternal B12 and offspring neurodevelopment was inconclusive, with variable findings across cognitive, language and motor domains.
A6. Antenatal diagnosis, supplementation and intervention								
Mearns et al. (76)	Auckland, New Zealand (Includes South Asian population)	South Asian women of childbearing age; age: 18–50 years; South Asian ethnicity; Auckland, New Zealand; not pregnancy-specific	3-arm RCT	62	NR	Serum B12 and HoloTC; B12 < 222 pmol/L and HoloTC < 45 pmol/L indicated insufficient/deficient status	B12 supplementation; biomarker response	Oral cyanocobalamin 6 μg/day increased serum B12 by 30% and HoloTC by 42% over 6 months; dietary advice alone did not significantly improve B12 biomarkers.
Siddiqua et al. (49)	Dhaka, Bangladesh	Anemic pregnant women; age: 18–35 years; 11–14 weeks' gestation; Hb < 11 g/dL; low-income community	Blinded placebo-controlled RCT	68	June 2010–Aug 2012	Plasma B12; deficiency < 150 pmol/L; marginal 150–220 pmol/L; MMA >271 nmol/L and tHcy >10 μmol/L assessed	Antenatal/postpartum B12 supplementation; maternal/infant/breast-milk B12 status and vaccine response	250 μg/day B12 improved maternal, breast-milk and infant B12 status, lowered MMA, and increased maternal H1N1-specific immune responses.
Devi et al. (79)	South India (Bangalore)	Pregnant women with low serum B12; age/ethnicity: NR	Randomized partially open-label intervention trial	93 (milk+B12 *n* = 30, milk+placebo *n* = 30, placebo *n* = 33)	NR	Serum B12; low < 200 pmol/L	B12/protein-energy supplementation; methionine remethylation and placental methylation	Combined 10 μg/day B12 + 500 mL milk/day showed a non-significant increase in methionine remethylation (P = 0.057); placental mRNA, LINE-1 and VEGF promoter methylation did not differ.
Wang et al. (68)	Turkey, USA and South India (component trials)	Vitamin B12-deficient adults; mean age across component trials: 38.6–72 years; not pregnancy-specific	Cochrane SR of 3 RCTs	153	1993–2010 across component trials	Serum B12; deficiency < 160 pg/mL in two trials and < 200 pg/mL in one trial	Route of administration (oral vs intramuscular vitamin B12)	Low-quality evidence indicated similar effects of oral and IM B12 on serum B12 normalization; oral treatment had lower costs.
Schroder et al. (61)	British Columbia, Canada	Pregnant women; age: NR; median gestation 11.4 and 16.1 weeks; ethnicity: 50% European and 50% South Asian; British Columbia, Canada	Retrospective cohort/diagnostic reference-interval study	723	NR	Serum total B12, HoloTC and MMA; reference limits: B12 < 89.9/ < 84.0 pmol/L, HoloTC < 29.5/ < 26.0 pmol/L, MMA >371/>374 nmol/L in 1st/2nd trimester; change-point thresholds: B12 < 186/ < 180 pmol/L, HoloTC < 62.2/ < 67.5 pmol/L	Pregnancy-specific B12 biomarker reference intervals/diagnostic assessment	Pregnancy-specific reference limits were established; B12, HoloTC and MMA reference limits differed by ethnicity, whereas change-point thresholds did not.
Chandyo et al. (74)	Nepal; community-based, 3 hospitals	Pregnant women; age: 20–40 years; ≤ 15 weeks' gestation; community-based recruitment through home visits/outpatient departments; ethnicity: NR	Community-based double-blind placebo-controlled RCT	800	28 Mar 2017–15 Oct 2020 (enrolment); follow-up to 18 May 2022	Plasma B12; low/marginal < 221 pmol/L	Maternal B12 supplementation; infant growth/neurodevelopment	50 μg/day B12 substantially improved maternal B12 status but did not improve infant length at 12 months or cognitive development at 6/12 months.
Finkelstein et al. (73)	India, Bangladesh, South Africa and Croatia	Pregnant women; age: generally18–35 years, although not all component trials reported age; ethnicity: NR; LMIC populations	Cochrane SR of 5 RCTs	984	NR	Maternal B12 assessed in component RCTs; deficiency < 150 pmol/L at enrolment; supplementation doses 5–250 μg/day	Antenatal B12 supplementation; maternal/infant B12 status and pregnancy/child outcomes	B12 supplementation may reduce maternal deficiency and increase maternal/infant B12 concentrations; evidence was very low certainty and longer-term clinical benefit remained uncertain.
Siddiqua et al. (77)	Dhaka, Bangladesh	Anemic pregnant women; age: 18–35 years; 11–14 weeks' gestation; Hb 7–11 g/dL; low-income community; ethnicity: NR	Blinded placebo-controlled RCT; secondary analysis	68	NR	Plasma B12 by ECLIA; no study-specific B12 deficiency cut-off reported	B12 supplementation; oxidative stress and inflammatory/immune markers	250 μg/day B12 reduced maternal and infant plasma 8-OHdG, altered cytokine profiles, and improved maternal and infant B12 status.
Sharma et al. (80)	Urban India	Anemic pregnant women attending urban antenatal clinics; age/ethnicity: NR	Open randomized trial	311	NR	Serum B12 assessed; deficiency cut-off NR	Antenatal supplementation with B12 + iron/folic acid; maternal hematological/biochemical outcomes	Adding 15 μg/day B12 via B-complex to iron–folic acid did not improve mean Hb or B12 versus iron–folic acid alone; both groups improved anemia, while the 5 mg folic acid arm showed a fall in B12.
Arora et al. (64)	New Delhi, India	Pregnant women; up to 14 weeks' gestation; attending antenatal clinic at Smt Sucheta Kriplani Hospital; age/ethnicity: NR	Descriptive cross-sectional study	100	Nov 2019–Oct 2021	Serum cobalamin by chemiluminescence; B12 deficiency < 200 pg/mL; HoloTC ≤ 23.78 ng/mL and MMA ≥11.67 ng/mL by ROC	Biomarker/diagnostic assessment	90% had B12 < 200 pg/mL; HoloTC and MMA showed potential as early markers; all 24 women above the MMA ROC cutoff had B12 deficiency.
Nagpal et al. (15)	New Delhi, India and Kathmandu, Nepal	Vegetarian pregnant women; age: 18–40 years; enrolled before 12 weeks' gestation; SBISR, New Delhi and PMWH, Kathmandu; ethnicity: NR	Multicentre double-blind RCT	531 completed	Oct 2018–Sep 2022	Maternal serum B12 deficiency < 197 pg/mL; HoloTC < 25.10 pmol/L; homocysteine ≥12 μmol/L with folate supplementation or ≥15 μmol/L without	Antenatal B12 supplementation; maternal and infant B12/neurodevelopment	250 μg/day methylcobalamin increased maternal B12 more than 50 μg/day and improved infant mental DQ at 9–12 months; motor DQ did not differ.
B. Evidence from outside South Asia								
Torsvik et al. (57)	Bergen, Norway	Infants with birth weight 2000–3000 g; mean gestational age: 37 weeks; maternal age/ethnicity: NR	Prospective cohort with nested double-blind RCT	80 infants in cohort; 32 randomized	Dec 2008–Apr 2010	Plasma cobalamin, total homocysteine and MMA; total homocysteine >6.5 μmol/L indicated biochemical signs of impaired cobalamin function and was used for RCT eligibility	Infant B12 supplementation; biochemical status and neurodevelopment	Among infants with elevated homocysteine, 400 μg hydroxycobalamin IM reduced homocysteine and MMA and improved gross-motor scores after 1 month vs. placebo.
Cai et al. (42)	Tianjin, China; Northern, Northwestern and Northeastern China	Chinese Han women with NTD-affected offspring and age-matched healthy controls; maternal age: NR	Case-control study	122 (61 cases, 61 controls)	November 2010–May 2014	Vitamin B12 not assessed; maternal plasma folate, homocysteine, S-adenosylmethionine and S-adenosylhomocysteine measured; one-carbon pathway gene polymorphisms genotyped	Maternal one-carbon metabolism and genetic polymorphisms; NTDs	Maternal folate status and polymorphisms in MTHFD1, MTHFR, MTRR and RFC1 were associated with NTD risk, supporting a role for one-carbon metabolism.
Jarquin Campos et al. (62)	Switzerland/Liechtenstein	General laboratory patient population; median age: 56 years (IQR 41–68); ethnicity: NR; not pregnancy-specific	Retrospective diagnostic accuracy study	11,833 samples from 9,464 patients	1 Dec 2006–19 Oct 2018	B12, holotranscobalamin (HoloTC), MMA (MMA) and homocysteine; 4cB12 ≤ -0.5 defined low/subclinical B12 status and ≤ -1.5 possible/probable deficiency; ROC-derived cut-offs: HoloTC < 45 pmol/L, B12 < 229 pmol/L, MMA >245 nmol/L, homocysteine >15 μmol/L	Biomarker/diagnostic accuracy	HoloTC showed the highest diagnostic accuracy for low/subclinical B12 status (AUC 0.92), followed by MMA (0.91), B12 (0.90) and homocysteine (0.78).
Zec et al. (75)	Split, Croatia	Healthy, non-anemic pregnant women recruited from eight gynecological primary-care practices; mean age: 31.08 ± 4.04 years; ethnicity: NR	RCT	200	NR	Vitamin B12 supplementation; no B12 deficiency cut-off reported	Antenatal B12 supplementation; hematological and biochemical outcomes	Adding 5 μg/day B12 for 100 days to standard iron and folic acid improved selected hematological/biochemical markers and reduced likelihood of anemia, although changes remained within reference ranges.
Li S. et al. (78)	Rural northwest China (Xunyi County, Shaanxi)	Pregnant women; age: 15–49 years; < 20 weeks' gestation; rural Xunyi County; newborns with available cord-blood samples; ethnicity: NR	Cluster-RCT; cord-blood substudy	331 mother–newborn pairs	January–December 2017	Cord plasma vitamin B12, folate and total homocysteine measured; no specific vitamin B12 deficiency cut-off reported	Antenatal vitamin B-complex supplementation; neonatal cord-blood vitamin B12 status and homocysteine	Antenatal vitamin B-complex supplementation was associated with higher cord plasma B12 and lower homocysteine in newborns.
Dib et al. (63)	Oxford, UK	Healthy pregnant women receiving antenatal care at John Radcliffe Hospital; mean age: 31.1 ± 3.52 years (range 22–39); ethnicity: NR	Diagnostic/biomarker cohort; longitudinal analysis of biobank samples	95 final analytic cohort (114 originally recruited)	14 Jul 2009–6 Jun 2011	Serum B12, HoloTC, homocysteine and MMA; B12 < 148 pmol/L; HoloTC < 32 pmol/L; MMA >350 nmol/L; cB12 < -0.5	Biomarker/diagnostic assessment	cB12 declined significantly in the third trimester (−66%, P < 0.001); trimester-specific reference ranges were identified, but no biomarker reliably predicted future B12 status.
Wirthensohn et al. (58)	Global	Infants	SR and analysis of published case reports/case series	292	NR	Infant serum vitamin B12; < 148 pmol/L probable deficiency; 148–258 pmol/L possible deficiency; >258 pmol/L unlikely deficiency	Infant neurodevelopment/clinical manifestations	Infant B12 deficiency was associated with neurological and hematological manifestations, including developmental delay, hypotonia, apathy/lethargy, irritability and movement disorders.
Rooney et al. (47)	Dublin, Ireland	Mother–child pairs from the ROLO cohort; secundigravida women >18 years; singleton pregnancy ≤ 18 weeks; ethnicity: NR	Secondary analysis of a longitudinal birth cohort	759	2007–2011	Dietary vitamin B12 intake assessed using 3-day food diaries in each trimester; dietary reference intake 1.5 μg/day	Dietary B12 intake; fetal growth, placental weight and birthweight	Maternal dietary B12 intake was not associated with fetal abdominal circumference or estimated fetal-weight trajectories, placental weight, or birthweight.
Kadam et al. (39)	Brooklyn, New York, USA	Pregnant women with and without GDM; recruited at 25–33 weeks' gestation; age/ethnicity: NR	Prospective observational cohort with 2-year follow-up	38 complete mother–child pairs	NR	Maternal dietary vitamin B12 intake assessed as part of one-carbon nutrient intake; maternal blood nutrient levels also assessed; no B12 deficiency cut-off reported	Maternal one-carbon nutrient intake; offspring growth and neurodevelopment	Higher maternal one-carbon nutrient intake was associated with lower child body weight at 2 years; higher food folate and choline intake was associated with better language, and folate with better cognitive scores.
Bonificacio et al. (56)	Multiple countries/populations	Pregnant women/mother–child pairs; component studies included supplementation trials and observational studies; age/ethnicity: NR	SR of 11 studies (4 RCTs, 7 observational)	11 studies	NR	Maternal B12 status/dietary intake/supplementation; definitions and cut-offs varied	Maternal B12 status/supplementation; child neurodevelopment/cognition	Maternal B12 supplementation was associated with cognitive/language effects up to 42 months; evidence was limited, particularly from RCTs.
Hrezova et al. (53)	Brno and Znojmo, Czech Republic	Mother–child pairs from the Czech ELSPAC birth cohort; age/ethnicity: NR	Population-based prospective longitudinal birth cohort	5,151 mother–child pairs; 854 children in 8-year IQ subcohort	1991–1992 enrolment; follow-up through childhood	Maternal dietary vitamin B12 intake estimated from a 145-item food-frequency questionnaire at 32 weeks' gestation; no B12 deficiency cut-off reported	Maternal dietary B12 intake; offspring speech/language development and intelligence	Higher prenatal B12 intake was associated with better language/talking-understanding at 18 months and higher verbal IQ at 8 years; associations were inconsistent at 5–7 years and fully adjusted total IQ was not significant.
Samavat et al. (38)	UK (Coventry)	Pregnant women; age: 25–38 years; ethnicity: Caucasian; paired abdominal subcutaneous and omental adipose tissue samples	Mechanistic study using human adipose tissue, *ex vivo* and *in vitro* models	115 participants	NR	Serum vitamin B12; deficiency defined as < 150 pmol/L	Metabolic mechanisms/adipose tissue metabolism	Maternal B12 deficiency was associated with dysregulated fatty-acid metabolism and increased adipose-tissue inflammatory responses.

B12, vitamin B12; CI, confidence interval; cB12, combined vitamin B12 status indicator; DQ, developmental quotient; ECLIA, electrochemiluminescence immunoassay; EPL, early pregnancy loss; FFQ, food-frequency questionnaire; GDM, gestational diabetes mellitus; Hb, hemoglobin; HOMA-IR, homeostatic model assessment of insulin resistance; HoloTC, holotranscobalamin; IM, intramuscular; IPD, individual participant data; IQR, interquartile range; LBW, low birth weight; MA, meta-analysis; MMA, methylmalonic acid; NR, not reported; NTD, neural tube defect; OR, odds ratio; RCT, randomized controlled trial; RoM, ratio of means; RPL, recurrent pregnancy loss; ROC, receiver operating characteristic; SAM, S-adenosylmethionine; SAH, S-adenosylhomocysteine; SGA, small for gestational age; SMD, standardized mean difference; SR, systematic review; tHcy, total homocysteine. Section A includes studies conducted in South Asia, studies enrolling South Asian participants, and global evidence syntheses that include South Asian populations. Section B contains comparator evidence from outside South Asia without a specific South Asian population focus. Study periods and B12 cut-offs are reported where available; NR indicates not reported.

### 2.5 Approach to synthesis and appraisal

The evidence was synthesized thematically rather than study by study. Findings from observational studies are reported as associations, mechanistic evidence is described separately, and evidence from randomized controlled trials is identified where applicable. To facilitate interpretation of the evidence, each major finding was linked to its primary evidence type and corresponding strength of inference in Table 2. The three predefined criteria for strength of inference were applied at the level of each major research question or evidence synthesis, rather than as a formal assessment of individual studies. Specifically, the assessment considered (i) the design of the strongest available evidence relevant to the question, (ii) the consistency of findings across independent studies and populations, and (iii) the extent to which residual confounding or other limitations constrained interpretation. These criteria were used to judge the overall strength of inference for each major finding and were not used as a separate risk-of-bias assessment of individual studies. Because no validated instrument exists for grading the heterogeneous mix of study designs encountered in this literature, and because a formal GRADE assessment was beyond the scope of this narrative review, the evidence categories presented in Table 1 were defined *a priori* using three explicit criteria. As this was a narrative review, the review protocol was not prospectively registered, and a formal risk-of-bias assessment of individual studies was not performed. These methodological limitations were considered when interpreting the findings and are discussed further in the Discussion.

**Table 2:** Summary of key conclusions, quantitative evidence, and strength of inference.

Key conclusion	Highest level of supporting evidence	Studies/total *n*	Cases/events	Pooled/reported effect estimate (95% CI)	Heterogeneity	Strength of inference
Vitamin B12 supplementation improves maternal vitamin B12 status; evidence for functional-marker improvement is limited	Cochrane SR of 5 RCTs (73); individual RCT (49)	5 RCTs; *n* = 984 overall; B12 concentration: 3 RCTs, *n* = 412; B12 deficiency: 2 RCTs, *n* = 272	NR	B12 concentration: MD 60.89 pmol/L (95% CI 40.86–80.92); B12 deficiency: RR 0.38 (95% CI 0.28–0.51); MMA reduction was reported in individual trials but not pooled	I^2^ = 85% for B12 concentration	Limited: Consistent biochemical improvement, but evidence was rated very low certainty and clinical benefits remain uncertain
Oral vitamin B12 is broadly comparable to intramuscular therapy for biochemical correction	Cochrane SR of RCTs (68)	3 RCTs; *n* = 153	—	Two trials found no meaningful difference; one trial favored oral therapy (MD 680 pg/mL, 95% CI 392.7–967.3)	Not pooled	Low: Small, heterogeneous trials with methodological limitations
Maternal vitamin B12 deficiency is associated with increased risk of gestational diabetes mellitus (GDM)	SR/MA of observational studies; 4 prospective cohorts (33)	10 studies; *n* = 10,595 overall; pooled cohort analysis: 4 studies, *n* = 7,243	NR	OR 1.46 (95% CI 1.21–1.79); South Asian subgroup OR 1.95 (95% CI 1.26–3.03)	I^2^ = 59.0% overall; South Asian subgroup I^2^ = 0%	Limited to moderate: Prospective cohort evidence supports an association, but overall evidence quality was low
Maternal vitamin B12 concentration is not significantly associated with GDM overall	SR/MA of observational studies (33)	6 studies	NR	OR 0.97 (95% CI 0.76–1.25)	I^2^ = 81.6%	Limited: No significant overall association and substantial heterogeneity
Maternal vitamin B12 deficiency in the second/third trimester is associated with higher risk of GDM	SR/MA of observational studies (34)	6 studies; *n* = 7,326	1,017 GDM; 6,309 non-GDM	OR 1.59 (95% CI 1.10–2.29); Asian subgroup OR 2.08 (95% CI 1.47–2.96)	I^2^ = 77%	Limited to moderate: Association was stronger in Asian populations, but heterogeneity was substantial
Lower maternal vitamin B12 concentration in later pregnancy is associated with GDM, particularly in Asian populations	SR/MA of 15 observational studies (34)	15 studies; *n* = 15,190 overall	NR	Overall MD −6.83 pmol/L (95% CI −17.56 to 3.89); second/third trimester MD −10.79 pmol/L (95% CI −21.37 to −0.21); Asian subgroup MD −25.78 pmol/L (95% CI −38.56 to −12.99)	I^2^ = 78%	Limited: Overall association was not significant; findings varied by gestational timing and population
A high folate:low vitamin B12 ratio in the second/third trimester is associated with higher risk of GDM	SR/MA of observational studies (34)	5 studies	NR	OR 1.87 (95% CI 1.46–2.41)	NR	Moderate: Consistent association across five studies supports consideration of folate and B12 jointly
Low maternal vitamin B12/high folate status is associated with adverse offspring metabolic outcomes	Prospective cohort; Pune Maternal Nutrition Study (37)	1 cohort; *n* = 700	NR	No pooled estimate; low B12/high folate was associated with greater offspring insulin resistance and adiposity	—	Preliminary: Evidence is primarily from one South Asian cohort
Low maternal vitamin B12 is associated with low birth weight	IPD MA of longitudinal cohort studies (44)	22 eligible studies; 11,993 observations; 18 studies, 11,216 observations in IPD MA	NR	B12 deficiency (< 148 pmol/L): aRR 1.15 (95% CI 1.01–1.31)	I^2^ = 5% for the B12-deficiency/LBW analysis	Moderate: Large IPD evidence base with low heterogeneity; residual confounding cannot be excluded
Low maternal vitamin B12 may be associated with preterm birth	IPD MA (44); prospective cohort (50)	Rogne: 18 studies, *n* = 11,216; Gerety: 1 cohort, *n* = 376	Gerety: 47 preterm births	Rogne: per 1-SD increase in B12, aRR 0.89 (95% CI 0.82–0.97); B12 deficiency: aRR 1.21 (95% CI 0.99–1.49). Gerety: aOR 3.33 (95% CI 1.29–8.21)	Rogne: I^2^ = 0% for continuous B12; 20% for deficiency	Limited to moderate: Direction of association is consistent, but the deficiency estimate from Rogne was imprecise
Low maternal vitamin B12 concentration is associated with neural tube defects (NTDs)	MA of 38 case-control studies (40)	38 studies; *n* = 6,614	2,316 NTD cases; 4,298 controls	SMD −0.23 (95% CI −0.32 to −0.14); folate-comparable analysis SMD −0.19 (95% CI −0.28 to −0.10)	I^2^ = 58.3%	Limited to moderate: Association persisted when maternal folate did not differ, but heterogeneity and publication bias limit inference
Maternal vitamin B12 supplementation may improve infant neurodevelopment	Single RCT (MATCOBIND; 15)	1 RCT; 708 randomized; 531 completed follow-up	—	Mental DQ: 103.7 vs. 101.7, mean difference ≈2.0 points, *P* = 0.007; motor DQ not significantly different	—	Preliminary: Single trial, active comparator, and short follow-up require independent replication
Evidence linking maternal vitamin B12 status/intake with offspring neurodevelopment is inconclusive	Scoping review (52)	19 studies; *n* = 23,825	—	No pooled estimate; associations varied across cognitive, language, and motor outcomes	—	Inconclusive: Evidence is heterogeneous and includes observational and intervention studies
HoloTC and other functional biomarkers show promise for assessment of maternal vitamin B12 status, but pregnancy-specific diagnostic validation remains limited	Diagnostic accuracy and pregnancy biomarker studies (61–64)	4 studies; individual *n* = 11,833 mixed population, 114/95 pregnant women, 723 pregnant women, 100 pregnant women	—	Jarquin Campos: HoloTC AUC 0.912; pregnancy studies established trimester-specific biomarker/reference intervals and exploratory diagnostic utility	—	Limited: Diagnostic performance is promising, but no validated pregnancy-specific threshold is established

aOR, adjusted odds ratio; aRR, adjusted risk ratio; B12, vitamin B12; CI, confidence interval; DQ, developmental quotient; GDM, gestational diabetes mellitus; HoloTC, holotranscobalamin; I^2^, I-squared statistic; IPD, individual participant data; LBW, low birth weight; MA, meta-analysis; MD, mean difference; MMA, methylmalonic acid; NR, not reported; NTD, neural tube defect; OR, odds ratio; RCT, randomized controlled trial; RR, risk ratio; SMD, standardized mean difference; SR, systematic review. Quantitative estimates are reported as presented in the cited SRs/MAs or primary studies. For SRs/MAs, Studies / total *n* refers to the overall evidence base when the conclusion is based on the overall review, or to the specific quantitative subgroup when a pooled estimate is derived from a restricted subset. Samples from different reviews or independent studies were not combined. NR indicates that the relevant value was not reported or could not be established; — indicates not applicable. Strength of inference follows the a priori framework described in Section 2.5 and considers study design, consistency, heterogeneity, population/setting differences, and potential residual confounding; these categories are not formal GRADE ratings.

The predefined evidence categories were applied as follows. High evidence denotes findings consistently supported by randomized controlled trials with clinically relevant, rather than solely biochemical, endpoints. No conclusion presented in this review met these criteria. Moderate evidence denotes a consistent association demonstrated in meta-analyses of observational studies or individual participant data analyses and replicated across more than one independent population, although residual confounding cannot be excluded in the absence of randomized evidence. Weak to low evidence denotes findings derived from a limited number of heterogeneous trials or observational studies reporting inconsistent directions of effect. Preliminary evidence denotes findings supported primarily by a single trial, a single cohort, or evidence that has not yet been independently replicated. The category Biologically plausible was reserved for mechanistic pathways supported by experimental or *in vitro* evidence in which corresponding clinical evidence from pregnant women was absent or indirect; this designation reflects a proposed biological mechanism rather than an established clinical effect. Where a finding fulfilled the criteria for more than one evidence category, the more conservative classification was assigned.

## 3 Results synthesis

A total of 59 studies were included in the narrative synthesis. The characteristics of the included studies are summarized in Table 1. The principal findings and corresponding strength of inference are summarized in Table 2.

### 3.1 Burden of maternal vitamin B_12_ deficiency and its determinants

Estimates of the global prevalence of vitamin B_12_ deficiency during pregnancy vary substantially depending on the diagnostic threshold, assay, and population studied. A meta-analysis of erythropoiesis-associated micronutrient deficiencies reported a pooled prevalence of approximately 17%, with regional estimates ranging from 4.1% to 48.7%, while an earlier synthesis using broader insufficiency cut-offs reported a prevalence closer to 25% (11, 12). This wide variation is informative because, in the absence of standardized pregnancy-specific thresholds, prevalence estimates are not directly comparable across studies, a limitation that recurs throughout the literature.

Within this broader context, South Asia bears a particularly high burden. Community-based data from rural South India reported vitamin B_12_ deficiency in approximately 55% of pregnant women, while retrospective cross-sectional studies from mixed urban and rural populations in India and neighboring countries have reported prevalence rates ranging from 40% to 70% (6, 13, 14). The MATCOBIND trial, which enrolled predominantly vegetarian women from India and Nepal, likewise reported a high prevalence of first-trimester deficiency, with a baseline median serum vitamin B_12_ concentration of approximately 185 pg/mL (15). Population-based biomarker surveys among non-pregnant women of reproductive age support these observations and are particularly informative because they reflect preconception nutritional status. In rural Haryana, 57.7% of women were vitamin B_12_ deficient, while a further 23.5% had marginal concentrations; corresponding estimates from southern India were 48.3% and 26.0%, respectively (16, 17). Collectively, these findings indicate that vitamin B_12_ deficiency is highly prevalent across South Asia and is frequently established before conception. Nevertheless, these prevalence estimates should be interpreted with caution because different studies used varying biochemical thresholds to define deficiency, limiting direct comparison across populations.

The factors contributing to this burden are largely structural. Cobalamin is obtained almost exclusively from animal-source foods and is essentially absent from unfortified plant foods; consequently, vegetarian and vegan dietary patterns confer a high baseline risk of deficiency (18, 19). In India, this dietary vulnerability is compounded by economic constraints that limit access to animal-source foods, low dietary diversity, poor awareness of vitamin B_12_ deficiency among both patients and healthcare providers, malabsorption associated with recurrent gastrointestinal infections, and the absence of vitamin B_12_ from public food-distribution programmes (6, 20). Genetic variation in one-carbon metabolism and cobalamin transport genes has also been investigated as a potential contributor to inter-individual differences in vitamin B_12_-related biomarkers, including homocysteine concentrations, in South Asian populations (21–23, 91). Together, dietary practices, socioeconomic factors, and genetic susceptibility contribute to the persistently high prevalence of vitamin B_12_ deficiency across the region, highlighting the need for population-level preventive strategies.

### 3.2 Associated maternal and offspring outcomes

This section summarizes the maternal and fetal/neonatal outcomes associated with low maternal vitamin B_12_ status. Maternal and offspring outcomes are discussed together because they frequently share common underlying pathways related to impaired one-carbon metabolism and are intrinsically linked through placental function. Most of the available evidence is observational and therefore supports associations rather than causality. Evidence from randomized controlled trials, where available, is identified separately, and the potential influence of co-existing micronutrient deficiencies and socioeconomic factors should be considered when interpreting the findings discussed below. Because folate and vitamin B_12_ function within the same one-carbon metabolic pathway, findings were interpreted in relation to folate status where it was reported; however, folate was not assessed consistently across studies. Among studies that assessed both nutrients, direct evaluation of a folate:B12 ratio or folate–B12 imbalance was relatively uncommon. Where examined, however, a low-B12/high-folate pattern or altered folate:B12 relationship was reported in relation to gestational diabetes, offspring metabolic outcomes, and recurrent pregnancy loss, whereas other studies reported folate and B12 separately without demonstrating a clear interaction.

Maternal anemia represents the most direct hematological consequence of vitamin B_12_ deficiency. Studies describe megaloblastic changes associated with deficiency; however, co-existing iron deficiency may result in a dimorphic blood picture that normalizes the mean corpuscular volume and masks macrocytosis. Consequently, vitamin B_12_ deficiency may remain undetected within antenatal screening programmes that primarily focus on iron deficiency (6, 24). Hypertensive disorders of pregnancy, including preeclampsia, have also been associated with low maternal vitamin B_12_ status in observational studies and meta-analyses. Dietary evidence further supports this relationship. In a matched case–control study of 440 pairs of Chinese women, higher dietary intakes of vitamin B_12_, folate, vitamin B6, methionine, and choline were each inversely associated with preeclampsia, with an adjusted odds ratio of 0.68 (95% CI 0.52–0.88) for the highest compared with the lowest quartile of vitamin B_12_ intake (25). However, analyses that model these nutrients simultaneously suggest that the association cannot be attributed to vitamin B_12_ alone. Among 1,178 women supplemented with folate and vitamin B_12_ from before conception, hyperhomocysteinaemia predicted pregnancy-induced hypertension only in those carrying the MTHFR CC genotype, while lower rather than higher folate concentrations independently predicted progression from pregnancy-induced hypertension to preeclampsia (26). Overall, these findings indicate that the observed associations may reflect broader disturbances in one-carbon metabolism rather than an effect of vitamin B_12_ concentration in isolation. Nevertheless, in the absence of intervention trials evaluating vitamin B_12_ supplementation for the prevention of preeclampsia, causality remains unestablished (27, 28).

Pregnancy loss and recurrent miscarriage have also been associated with low maternal vitamin B_12_ status and elevated homocysteine concentrations. Several North Indian case–control studies have reported an increased risk among women with low vitamin B_12_ and high folate status. A clinical study combined with a meta-analysis reached a similar conclusion, reporting that low vitamin B_12_ and hyperhomocysteinaemia were each independent risk factors for early pregnancy loss and that their coexistence was associated with an odds ratio of 4.98 (*P* = 0.002), whereas folate deficiency alone was not associated with increased risk (29). These findings should be interpreted cautiously because potential nutritional and socioeconomic confounding cannot be excluded, and the case–control design does not establish whether the observed biochemical abnormalities preceded pregnancy loss (29–32). Thus, although some studies suggest a combined low-B12/high-folate pattern, the available evidence is insufficient to establish a consistent folate–B12 interaction for pregnancy loss.

Metabolic outcomes represent an important component of the evidence, particularly in South Asian populations. Observational studies suggest an association between low maternal vitamin B_12_ status, especially when accompanied by high folate concentrations, and an increased risk of gestational diabetes mellitus, as well as greater offspring insulin resistance and adiposity during childhood, as reported in the Pune Maternal Nutrition Study. Two meta-analyses further support the association between vitamin B_12_ status and gestational diabetes. Pooling data from 10 studies involving 10,595 women, vitamin B_12_ deficiency was associated with an odds ratio of 1.46 (95% CI 1.21–1.79) (33), while a high folate-to-vitamin B_12_ ratio was associated with an odds ratio of 1.87 (95% CI 1.46–2.41) (34).

The contribution of folate to this relationship was not consistent across studies. A dose–response meta-analysis of 13 observational studies reported that higher red blood cell folate concentrations were associated with an increased risk of gestational diabetes with moderate-certainty evidence, whereas the association with plasma or serum vitamin B_12_ did not reach statistical significance and was graded as very low-certainty evidence (35). In a study of British-Bangladeshi women, total vitamin B_12_ and holotranscobalamin concentrations were inversely correlated with third-trimester HOMA-IR. However, vitamin B_12_ concentrations did not differ significantly between women with and without gestational diabetes, and the correlation between total vitamin B_12_ and holotranscobalamin was weak, highlighting the complexity of interpreting individual biomarkers of vitamin B_12_ status (36).

The evidence linking maternal vitamin B_12_ status with childhood metabolic outcomes remains limited and is derived predominantly from a small number of cohorts. In the Pune Maternal Nutrition Study, maternal vitamin B_12_ deficiency, particularly in combination with high folate status, was associated with increased adiposity and insulin resistance in the offspring during childhood (37). More recently, a small prospective follow-up study involving only 38 mother–child pairs reported that higher maternal intake of one-carbon nutrients during pregnancy was associated with lower child body weight and improved language and cognitive scores at two years of age (38, 39). Although these findings are biologically plausible, the limited sample size and lack of independent replication substantially restrict the strength of the available evidence. Consequently, while maternal vitamin B_12_ deficiency and disturbed one-carbon metabolism have emerged as potential contributors to adverse metabolic programming in offspring, the limited number of studies prevents determination of whether these associations reflect vitamin B_12_ deficiency itself, high folate exposure, or their combined metabolic effect, and further large, prospective studies are required before firm conclusions can be drawn.

Among congenital outcomes, the association between periconceptional vitamin B_12_ deficiency and neural tube defects (NTDs) is one of the most clinically important. A meta-analysis of 38 case–control studies reported lower maternal vitamin B_12_ concentrations in NTD-affected pregnancies, apparently independent of folate status (40). Related congenital anomalies, including orofacial clefts, have also been associated with low maternal vitamin B_12_ status in case–control studies from Tamil Nadu and cross-sectional studies from Bangladesh (92, 93). An earlier meta-analysis of 32 studies involving 1,890 mothers of NTD-affected pregnancies and 3,995 controls similarly reported modestly lower maternal vitamin B_12_ concentrations (ratio of means 0.91, 95% CI 0.87–0.95), together with higher homocysteine and lower folate concentrations (41). Thus, although lower maternal B1_2_ appears to be associated with NTD risk even after consideration of folate, the broader evidence supports interpreting B1_2_ within the combined one-carbon metabolic context. Case–control studies incorporating genetic analyses further support this interpretation, with maternal folate concentrations and variants in MTHFR, MTHFD1, MTRR, and RFC1 all associated with NTD risk (42).

Low maternal vitamin B_12_ status has also been associated with impaired fetal growth and shorter gestational duration. Multiple observational cohort studies and an individual participant data meta-analysis have linked low maternal vitamin B_12_ concentrations with reduced fetal growth and low birth weight. Furthermore, growth-restricted infants born to vitamin B_12_-deficient mothers have been reported to exhibit reduced lean mass with relative adiposity, a body composition pattern associated with increased cardiometabolic risk later in life, although this observation requires confirmation in larger prospective studies (43–45).

The evidence, however, is not entirely consistent, and these inconsistencies are themselves informative. In the MAASTHI birth cohort from urban Bengaluru, in which 48.5% of women had low plasma vitamin B_12_ concentrations and 73.6% had elevated methylmalonic acid concentrations, vitamin B6 deficiency and impaired folate status, rather than vitamin B_12_ deficiency, were associated with low birth weight (46). Similarly, the ROLO cohort in Ireland reported no association between maternal dietary vitamin B_12_ intake and fetal growth trajectories, placental weight, or birth weight (47). An Indian cohort further suggested that alterations in the homocysteine pathway, rather than vitamin B_12_ concentration itself, were associated with low birth weight and neonatal hyperbilirubinaemia (48). These null and discordant findings suggest that the relationship between maternal vitamin B_12_ status and fetal growth is neither universal nor independent of the broader micronutrient and metabolic environment. In particular, the MAASTHI findings indicate that impaired folate status may contribute independently to fetal growth outcomes, whereas other South Asian evidence suggests that the combination of low B1_2_ and relatively high folate may also be relevant. Consequently, caution is warranted when generalizing findings from South Asian populations, where the association appears strongest, to other settings. Preliminary evidence from prospective cohort studies also suggests that low maternal vitamin B_12_ status may be associated with preterm birth (44, 49, 50).

Emerging evidence further suggests that maternal vitamin B_12_ status may influence offspring neurodevelopment, particularly cognitive, language, and motor development, although robust evidence from randomized controlled trials remains limited. A recent scoping review reported associations across these developmental domains while emphasizing the limited and heterogeneous nature of the available intervention evidence. Likewise, a Czech birth cohort observed that higher prenatal vitamin B_12_ intake was associated with improved child speech and intelligence quotient scores. More recently, the preconceptional PRIYA trial demonstrated improved offspring neurodevelopment at two years of age, while the MATCOBIND trial reported improved infant neurodevelopment at nine months (15, 51–53). However, folate status or the folate-to-vitamin B_12_ relationship was not consistently reported across these studies, limiting assessment of whether the observed neurodevelopmental associations were independent of maternal folate status.

The randomized evidence, however, is less consistent than these findings alone might suggest. In a South Indian trial administering 50 μg/day oral vitamin B_12_ from the first trimester until six weeks postpartum, no significant differences were observed across any Bayley developmental subscale at nine months of age. Follow-up assessment of the same cohort at 30 months demonstrated higher expressive language scores among supplemented children (β = 0.14, *P* = 0.03), whereas no improvements were observed in other developmental domains (54, 55). Notably, elevated maternal homocysteine concentrations, rather than the assigned vitamin B_12_ supplementation, showed the more consistent inverse association with expressive language and motor development. Similarly, a systematic review of 11 studies concluded that maternal vitamin B_12_ supplementation may influence cognitive and language development up to 42 months of age but noted that this conclusion was based on only four randomized controlled trials and therefore considered the evidence insufficient to support firm conclusions. A more recent scoping review of 19 publications likewise described the overall relationship as inconclusive (52, 56).

Where a clear benefit has been demonstrated, it has largely been confined to infants with established biochemical deficiency rather than unselected populations. In a randomized trial of infants with elevated homocysteine concentrations at six months of age, hydroxocobalamin supplementation reduced homocysteine and methylmalonic acid concentrations while significantly improving gross motor scores within one month (57). Taken together, these findings suggest that any beneficial effect of vitamin B_12_ on neurodevelopment is most apparent in deficient individuals, may preferentially affect specific language-related domains, and becomes more evident with longer follow-up. At the same time, the relatively small number of trials, limited sample sizes, and possibility of selective outcome reporting warrant cautious interpretation.

These concerns extend into infancy because breast milk vitamin B_12_ concentrations closely reflect maternal vitamin B_12_ status. Consequently, exclusively breastfed infants born to vitamin B_12_-deficient mothers may develop symptomatic deficiency during the first year of life, presenting with failure to thrive, megaloblastic anemia, hypotonia, and neurological regression. Importantly, severe neurological injury may not be completely reversible despite appropriate treatment (58, 59).

### 3.3 Diagnosis: implications for a screening pathway

Diagnosis is discussed here only in the context of developing a practical screening pathway. The principal challenge is that serum cobalamin, the most widely available test, declines physiologically during pregnancy and reflects the circulating rather than the intracellular vitamin B_12_ pool. Consequently, it may fail to detect early or functional deficiency, and universally accepted pregnancy-specific reference ranges remain unavailable (60, 61).

Functional biomarkers are generally considered to reflect tissue vitamin B_12_ status more accurately. Holotranscobalamin, the metabolically active fraction, has performed better than total serum vitamin B_12_ in mixed non-pregnant populations, although this advantage has not yet been established during pregnancy because validation studies remain limited and no universally accepted pregnancy-specific cut-off has been defined. A first-trimester Indian study found that nine of every ten women with normal serum vitamin B_12_ concentrations had low holotranscobalamin despite normal methylmalonic acid concentrations. This finding may indicate earlier detection of vitamin B_12_ depletion or may simply reflect the physiological decline in binding proteins during pregnancy; without an appropriate functional reference standard, these possibilities cannot be distinguished. Methylmalonic acid is more specific for functional vitamin B_12_ deficiency but may also be elevated in renal impairment, which is not uncommon in preeclampsia, and is not widely available in routine antenatal care in India (62–64).

Contemporary clinical recommendations broadly reflect this staged diagnostic approach. Recent Australian and UK guidelines recommend holotranscobalamin as a first-line or sequential assessment during pregnancy, with methylmalonic acid or homocysteine used to confirm functional deficiency where appropriate (65, 66). In clinical practice, vitamin B_12_ deficiency is frequently overlooked because its early manifestations overlap with those of normal pregnancy, and neurological symptoms may develop before anemia becomes apparent. In resource-limited settings, knuckle hyperpigmentation may provide a useful, inexpensive clinical clue, as it can accompany or even precede hematological manifestations (67). The key diagnostic features relevant to a screening pathway are summarized in Table 3.

**Table 3:** Diagnostic biomarkers of vitamin B12 status in pregnancy.

Biomarker	Normal range	Pregnancy considerations	Advantages and limitations	Clinical role
Serum vitamin B12 (total cobalamin)	>300 pg/mL (>221 pmol/L)	Declines by 10–30% during pregnancy and may underestimate deficiency	Widely available and inexpensive; reflects circulating vitamin B12 and may miss functional deficiency	Initial screening test
Holotranscobalamin (holoTC)	>35 pmol/L	Declines before total vitamin B12; recommended for early assessment where available	Falls before total vitamin B12; the AUC of ~0.92 derives from a mixed non-pregnant population and has not been reproduced in pregnancy; limited availability and no validated pregnancy-specific cut-offs	Early-detection marker where available; not yet validated for routine antenatal use
Methylmalonic acid (MMA)	<0.28 μmol/L	Increases in functional deficiency despite normal serum vitamin B12	Specific marker of intracellular deficiency; affected by renal impairment and not routinely available	Confirmatory test
Total homocysteine (tHcy)	<12 μmol/L	Influenced by vitamin B12 and folate status; declines slightly during normal pregnancy	Sensitive functional marker but lacks specificity and is influenced by folate and vitamin B6	Adjunctive functional marker

AUC, area under the ROC curve. Adapted from NICE NG239 (65); Jarquin Campos et al. (62); MBS Online (66).

### 3.4 Supplementation and safety: the randomized evidence

Where vitamin B_12_ deficiency is confirmed, high-dose oral cobalamin (1,000–2,000 μg/day) is an effective, inexpensive, and well-tolerated first-line treatment for most pregnant women because pharmacological doses are absorbed through passive diffusion independent of intrinsic factor. Available evidence suggests that oral vitamin B_12_ is comparable to intramuscular therapy for biochemical correction, although this conclusion is based on a relatively small number of heterogeneous trials. This is particularly relevant in settings where parenteral administration may be limited by restricted access to health facilities, availability of trained personnel, and cold-chain requirements (68–70).

Parenteral hydroxocobalamin is generally reserved for women with severe megaloblastic anemia, overt neurological disease, or malabsorption. Vitamin B_12_ has a well-established safety profile, and no tolerable upper intake level has been established by major regulatory bodies because of its saturable active absorption and efficient renal clearance. Supplementation trials using both physiological and pharmacological doses have not reported teratogenic effects or adverse maternal outcomes. The three principal forms of vitamin B_12_–cyanocobalamin, methylcobalamin, and hydroxocobalamin—are all considered safe and effective. The preference for methylcobalamin in some pregnancy protocols is based primarily on mechanistic considerations rather than demonstrated clinical superiority (71–73).

The findings from randomized trials are generally consistent but should be interpreted cautiously. Randomized studies consistently demonstrate improvements in biochemical markers, including serum vitamin B_12_, methylmalonic acid, and homocysteine, across different study populations. However, evidence for corresponding clinical benefits has been considerably less consistent. The Cochrane review of five randomized trials concluded that vitamin B_12_ supplementation may improve maternal and infant vitamin B_12_ status, although the certainty of the evidence was very low and evidence for longer-term clinical benefits remains insufficient (73). Similarly, a large community-based trial conducted in Nepal reported improved maternal vitamin B_12_ status following supplementation but found no differences in infant length-for-age or cognitive development at 12 months compared with placebo, suggesting that correction of maternal vitamin B_12_ status alone may be insufficient to improve these outcomes within the available follow-up period (74).

The wider randomized literature consistently demonstrates biochemical improvement across different doses, populations, and routes of administration. Daily doses as low as 5–6 μg improved vitamin B_12_ biomarkers in non-anemic Croatian women and South Asian women of childbearing age living in New Zealand, increasing serum vitamin B_12_ by 30% and holotranscobalamin by 42% after six months of supplementation (75, 76). In Bangladesh, supplementation with 250 μg/day increased plasma, colostrum, and breast milk vitamin B_12_ concentrations, reduced neonatal and infant methylmalonic acid concentrations, lowered an oxidative stress marker, and improved vaccine-specific antibody responses in mothers (49, 77). Likewise, a cluster-randomized trial conducted in rural China reported higher cord blood vitamin B_12_ concentrations and lower homocysteine concentrations among newborns of women receiving a vitamin B-complex supplement (78).

Two trials further illustrate the limitations of relying solely on biochemical endpoints. In South Indian women with low vitamin B_12_ status, supplementation with 10 μg/day vitamin B_12_, with or without balanced protein–energy supplementation, resulted only in a non-significant trend toward greater methionine remethylation and produced no detectable changes in placental LINE-1 or VEGF promoter methylation (79). Similarly, among anemic pregnant women in India, the addition of 15 μg vitamin B_12_ to iron and folic acid supplementation did not improve hemoglobin concentrations or mean vitamin B_12_ levels beyond those achieved with iron and folic acid alone, although women receiving 5 mg folic acid demonstrated a reduction in vitamin B_12_ concentrations (80).

The MATCOBIND trial randomized 708 predominantly vegetarian women from India (*n* = 370) and Nepal (*n* = 338), of whom 531 completed follow-up. Participants received either 250 μg/day methylcobalamin or a 50 μg/day active comparator from the first trimester until six months postpartum. Supplementation improved maternal and infant vitamin B_12_ status and was associated with improved infant neurodevelopment at nine months (15). Nevertheless, these findings should be interpreted within the context of the study design. MATCOBIND represents a single trial, follow-up was limited to infancy, the comparator was a low-dose active control rather than placebo, and independent replication remains necessary before firm conclusions regarding neurodevelopmental benefit can be drawn (15).

Overall, the available randomized evidence demonstrates consistent biochemical improvement but less consistent evidence for meaningful clinical benefit. Biochemical correction should therefore not be assumed to translate directly into improved maternal or offspring health outcomes. The available evidence further suggests that the timing of intervention may be important, with preconception supplementation and broader nutritional support potentially offering greater benefit. This interpretation is supported by the improved offspring neurodevelopment reported in the preconceptional PRIYA trial at two years of age (51).

## 4 Discussion

### 4.1 Principal findings

Overall, the available evidence supports three principal conclusions. First, maternal vitamin B_12_ deficiency is highly prevalent in South Asia and is driven by persistent factors, including dietary practices, socioeconomic constraints, and genetic susceptibility, rather than by transient influences. Second, low maternal vitamin B_12_ status is associated with a wide range of adverse maternal and offspring outcomes that appear to converge on a limited number of shared biological mechanisms involving disturbed one-carbon metabolism, impaired methylation capacity, and altered placental function. However, the available evidence is derived predominantly from observational studies and therefore demonstrates associations rather than causality. Third, although vitamin B_12_ supplementation consistently improves biochemical markers of deficiency, evidence that these improvements translate into clinically meaningful maternal and offspring benefits remains limited across randomized trials. The persistent gap between biochemical correction and demonstrable clinical benefit is an important consideration when interpreting the current evidence and should be taken into account when making clinical or policy recommendations.

### 4.2 Findings in the context of the existing literature

The prevalence estimates synthesized in this review are consistent with earlier evidence once differences in diagnostic thresholds are taken into account, and the apparent disagreement between published estimates is largely methodological rather than biological. The most recent global meta-analysis reported a pooled prevalence of maternal vitamin B_12_ deficiency of 17.1% using a deficiency cut-off, whereas the earlier synthesis by Sukumar and colleagues reported vitamin B_12_ insufficiency in 21%, 19%, and 29% of women during the first, second, and third trimesters, respectively, using a higher insufficiency threshold (11, 12). Neither estimate is directly comparable with the 40–70% prevalence consistently reported from Indian populations because these studies generally apply a deficiency threshold of < 200 pg/mL in populations characterized by high rates of vegetarianism, low dietary diversity, and limited intake of animal-source foods. The higher burden observed in South Asia therefore appears genuine, although its magnitude is amplified by the continued absence of universally accepted pregnancy-specific diagnostic thresholds. This is not simply a methodological issue but has important clinical and public health implications because any prevalence-based argument supporting national supplementation policies depends on a denominator that remains inconsistently defined. Consequently, although the available evidence strongly supports the identification and treatment of deficient women, it does not yet justify universal vitamin B_12_ supplementation during pregnancy.

The biological findings provide a coherent framework for interpreting the associations observed across maternal and offspring outcomes. Vitamin B_12_ functions as a cofactor for methionine synthase, linking cobalamin status to folate-dependent remethylation of homocysteine and to the generation of methionine and downstream methyl donors. Impairment of this pathway can result in a functional folate trap, in which 5-methyltetrahydrofolate becomes metabolically unavailable, potentially reducing nucleotide synthesis and affecting rapidly dividing tissues such as haematopoietic precursors, trophoblast, and embryonic neuroepithelium (24, 81). This provides a plausible biological basis for the observed associations with megaloblastic anemia and some congenital outcomes.

A second pathway involves accumulation of homocysteine when remethylation is impaired. Experimental evidence suggests that elevated homocysteine may contribute to oxidative stress, impaired nitric oxide bioavailability, and endothelial dysfunction, providing a potential mechanistic link between disturbed one-carbon metabolism and placental or vascular complications such as preeclampsia (82, 83). However, the inconsistent clinical effects of interventions aimed at lowering homocysteine indicate that homocysteine should not necessarily be regarded as the sole causal mediator; it may instead represent an intermediate marker of broader metabolic disturbance (84).

A third potential pathway involves methylation. Because one-carbon metabolism contributes to the generation of S-adenosylmethionine (95), vitamin B_12_ deficiency may influence the cellular methylation environment during critical periods of embryonic and fetal development, including DNA and histone methylation (94). Such changes could provide a mechanism through which maternal nutritional status influences longer-term offspring phenotype. Nevertheless, the direction, persistence, tissue specificity, and clinical significance of these epigenetic alterations remain uncertain, and therefore this pathway should be considered biologically plausible rather than established as a causal explanation for the observed clinical associations (85).

Taken together, these mechanisms suggest that the clinically relevant exposure may often be disturbed one-carbon metabolism rather than circulating vitamin B_12_ concentration in isolation. This distinction is particularly important when interpreting studies in which folate, homocysteine, vitamin B_12_, and other one-carbon nutrients differ simultaneously, because an observed association with vitamin B_12_ may partly reflect the broader metabolic environment.

Perhaps the most important finding emerging from this synthesis is the persistent disconnect between biochemical repletion and demonstrable clinical benefit. While randomized trials consistently report improvements in biochemical markers such as serum vitamin B_12_, methylmalonic acid, and homocysteine, corresponding improvements in maternal or offspring clinical outcomes have been considerably less consistent. This apparent inconsistency may partly reflect differences in the timing of intervention, baseline nutritional status, comparator groups, and duration of follow-up. The more favorable findings from preconceptional supplementation and from MATCOBIND, compared with several first-trimester trials, suggest that both the timing of supplementation and the developmental stage at which outcomes are assessed may influence the ability to detect clinical benefit (15, 51).

An equally important observation is that maternal homocysteine concentrations appear to predict offspring outcomes more consistently than assigned vitamin B_12_ treatment. This finding suggests that the intermediate metabolic state may represent a more biologically relevant exposure than circulating vitamin B_12_ concentration alone. Supporting this interpretation, supplementation with 10 μg/day vitamin B_12_ failed to produce significant changes in methionine remethylation or placental methylation markers among women with low vitamin B_12_ status (79). Together, these findings indicate that conventional supplementation doses may improve circulating biomarkers without sufficiently modifying the underlying one-carbon metabolic pathways through which clinical benefit is hypothesized to occur. This interpretation is also consistent with the mechanistic evidence presented throughout this review, which identifies disturbed one-carbon metabolism, rather than vitamin B_12_ deficiency alone, as the common biological pathway linking many of the observed maternal and offspring outcomes.

The low vitamin B_12_-high folate hypothesis, which is particularly relevant within the Indian antenatal care context, receives partial rather than unequivocal support from the available literature. The association between an imbalanced vitamin B_12_-folate status and gestational diabetes has been reproduced across multiple observational studies, with pooled analyses reporting an increased risk associated with low vitamin B_12_ in combination with high folate concentrations (33, 34). Similarly, the association with small-for-gestational-age infants has been demonstrated directly among South Indian women receiving routine high-dose folic acid supplementation (86). Nevertheless, the evidence does not conclusively attribute these outcomes to vitamin B_12_ deficiency alone. A dose–response meta-analysis found that higher red blood cell folate concentrations were independently associated with gestational diabetes with moderate-certainty evidence, whereas the corresponding evidence for vitamin B_12_ was graded as very low certainty (35). Furthermore, a large real-world cohort reported that lower rather than higher folate concentrations independently predicted progression from pregnancy-induced hypertension to preeclampsia (26). Across the broader outcome literature, however, folate was not consistently measured, adjusted for, or analyzed jointly with vitamin B_12_. Where both nutrients were considered, findings varied by outcome: some studies supported an association with a low-B1_2_/high-folate pattern or a higher folate-to-B1_2_ ratio, whereas other evidence, including that for neural tube defects, suggested that maternal vitamin B_12_ remained relevant after consideration of folate. Collectively, these findings suggest that disturbance of the one-carbon metabolic pathway, irrespective of whether it arises from vitamin B_12_ deficiency, folate imbalance, or their interaction, may represent the biologically relevant exposure. Accordingly, the practical implication is not that folic acid supplementation should be reduced or withdrawn, but rather that folic acid supplementation should ideally be accompanied by assessment of vitamin B_12_ status in populations where deficiency is common. At the same time, reducing folate provision would be difficult to justify in populations where red blood cell folate insufficiency remains common and the predicted prevalence of neural tube defects remains high (16, 17). Future studies should therefore measure and analyse maternal folate and vitamin B_12_ status jointly, rather than treating either nutrient in isolation. Prospective studies incorporating absolute concentrations as well as folate ratios, together with homocysteine and other functional biomarkers, would help determine whether adverse outcomes are associated with vitamin B_12_ deficiency itself, relative folate excess, or disruption of one-carbon metabolism as a whole.

The null findings deserve greater consideration than they have typically received within this literature. The MAASTHI cohort from urban Bengaluru, despite documenting low vitamin B_12_ concentrations in 48.5% of women and elevated methylmalonic acid concentrations in 73.6%, identified vitamin B6 deficiency and impaired folate status, rather than vitamin B_12_ deficiency, as the nutritional factors associated with low birth weight (46). Similarly, the ROLO cohort in Ireland reported no association between maternal vitamin B_12_ intake and fetal growth trajectories, placental weight, or birth weight (47). Rather than weakening the overall evidence base, these findings help define the circumstances under which vitamin B_12_ deficiency may be clinically relevant.

Two explanations appear compatible with the available evidence. First, the relationship between maternal vitamin B_12_ status and adverse pregnancy outcomes may depend on the severity and duration of deficiency, becoming clinically detectable only in populations with long-standing preconception deficiency. This interpretation is consistent with the strongest associations being observed in Indian cohorts such as the Pune Maternal Nutrition Study and the Mysore cohorts, where vitamin B_12_ deficiency is highly prevalent before conception and persists throughout pregnancy (37). Second, vitamin B_12_ concentrations may act as a marker of overall dietary quality, socioeconomic status, or broader nutritional adequacy rather than representing an independent causal determinant of pregnancy outcomes. This possibility cannot be excluded by observational studies and is supported by the largely null findings of randomized trials evaluating fetal growth and several clinical outcomes. Distinguishing between these competing explanations will require future intervention studies stratified according to baseline vitamin B_12_ status rather than further observational replication alone. Such an approach would help determine whether supplementation primarily benefits women with established deficiency or whether broader population-level effects can also be demonstrated.

The diagnostic conclusions of this review are intentionally more cautious than some current clinical recommendations. Recent Australian and United Kingdom guidance recommends holotranscobalamin as a first-line or staged assessment during pregnancy, with methylmalonic acid or homocysteine used to confirm functional deficiency where appropriate (65, 66). Outside pregnancy, the diagnostic performance of holotranscobalamin is well established (62). However, the pregnancy-specific evidence base remains comparatively limited and consists largely of studies establishing reference intervals or evaluating combinations of biomarkers rather than validating clinically meaningful thresholds against functional outcomes (61, 63, 64). Consequently, extrapolating diagnostic thresholds from non-pregnant populations may contribute to the same uncertainty that currently surrounds interpretation of total serum vitamin B_12_ concentrations during pregnancy.

For this reason, the present review supports the use of holotranscobalamin as an early indicator of vitamin B_12_ depletion where the assay is available but considers further validation of pregnancy-specific diagnostic thresholds an essential prerequisite before widespread implementation within routine antenatal screening programmes. This position lies between the recommendations of Behere and colleagues, who advocated inclusion of vitamin B_12_ within the national antenatal supplementation programme, and the conclusions of the recent Cochrane review, which judged the certainty of evidence supporting maternal and infant benefit to be very low (6, 73). The difference is therefore one of threshold for action rather than interpretation of the underlying evidence. The observational evidence is sufficiently consistent to support identification and treatment of women with confirmed vitamin B_12_ deficiency, whereas the current randomized evidence remains insufficient to justify universal supplementation of all pregnant women irrespective of baseline nutritional status.

### 4.3 Implications for antenatal practice and policy in South Asia

Despite these limitations, the available evidence is sufficient to support targeted action in high-risk settings without waiting for universal policy mandates. A pragmatic approach comprises three components. First, women at increased risk including those following vegetarian or vegan diets, those from low-income households with limited dietary diversity, those with a history of adverse pregnancy outcomes, and those with suggestive clinical features should undergo risk-based screening at the first antenatal visit. Holotranscobalamin may be used where available, with serum vitamin B_12_ as an alternative, followed by confirmatory testing where indicated (6, 64). Second, women with confirmed vitamin B_12_ deficiency should receive prompt treatment with high-dose oral methylcobalamin or cyanocobalamin (1,000–2,000 μg/day), followed by repeat biochemical assessment after 6–8 weeks to evaluate treatment response (5). Third, supplementation should continue throughout lactation, and infant vitamin B_12_ status should be monitored in cases of severe maternal deficiency because exclusively breastfed infants of deficient mothers are at greatest risk of developing symptomatic deficiency (58).

This approach could be integrated into India's existing antenatal care programmes, including the Pradhan Mantri Surakshit Matritva Abhiyan and the Reproductive and Child Health Programme, both of which already provide structured antenatal visits and routine iron–folic acid supplementation without requiring major structural modifications (87, 88). The first antenatal visit, typically occurring between 8 and 12 weeks of gestation, provides an appropriate opportunity for risk stratification. Accredited Social Health Activist (ASHA) workers could undertake brief dietary assessments to identify women who may benefit from facility-based screening, while oral vitamin B_12_ supplementation could be incorporated into the existing iron–folic acid distribution system for women identified as being at high risk (6, 89). Capacity-building requirements are relatively modest and include improving awareness that vitamin B_12_ deficiency is common among vegetarian pregnant women, recognizing knuckle hyperpigmentation and neurological symptoms as potential clinical indicators, and promoting awareness that oral vitamin B_12_ therapy is both safe and effective. Nevertheless, laboratory capacity for vitamin B_12_ testing is likely to vary across healthcare settings and should be evaluated before wider implementation. A phased strategy therefore appears appropriate, beginning with pilot programmes for targeted screening and supplementation in high-burden states, followed by rigorous evaluation of programme coverage, biochemical and clinical outcomes, cost-effectiveness, and implementation feasibility before broader policy adoption is considered (8, 90).

### 4.4 Research gaps and future directions

Several important knowledge gaps remain. First, universally accepted pregnancy-specific diagnostic thresholds for vitamin B_12_ and its functional biomarkers are still lacking. Current thresholds are largely derived from non-pregnant adults and require validation in pregnancy-specific cohorts that account for the physiological decline in serum vitamin B_12_ concentrations together with gestational changes in methylmalonic acid and homocysteine concentrations.

Second, evidence supporting causality remains limited. The current literature is dominated by observational studies and relatively short-term intervention trials, highlighting the need for adequately powered, prospectively registered randomized controlled trials with follow-up extending from the periconceptional period into childhood. Although the MATCOBIND trial provides encouraging evidence regarding maternal vitamin B_12_ status and early infant neurodevelopment, follow-up to nine months is insufficient to evaluate longer-term neurodevelopmental outcomes, and independent replication remains essential before firm conclusions can be drawn.

Third, the question of universal vs. targeted supplementation remains unresolved. Universal supplementation would require convincing evidence of benefit across populations with differing baseline vitamin B_12_ status, together with robust evidence regarding long-term safety, programme feasibility, cost-effectiveness, and the optimal dose and duration of supplementation. At present, these requirements have not been met. By contrast, targeted supplementation of women at high risk of deficiency is supported by stronger evidence and is likely to represent the more feasible public health strategy. Accordingly, cost-effectiveness studies evaluating risk-based screening and targeted supplementation in South Asian populations should be prioritized.

Finally, food fortification of staple foods, including wheat flour and rice, should be evaluated as a complementary population-level strategy alongside individual supplementation, building on the public health experience gained through folate fortification programmes for neural tube defect prevention.

### 4.5 Strengths and limitations

A major strength of this review is its explicit question-driven approach and the transparent mapping of each principal conclusion to the strength of the supporting evidence (Table 2). This enables readers to interpret the findings within the context of the quality and consistency of the underlying evidence rather than assigning equal confidence to all reported associations.

Several limitations should also be acknowledged. First, the evidence base for most clinical outcomes remains predominantly observational and is therefore susceptible to residual confounding arising from co-existing micronutrient deficiencies, socioeconomic status, and overall dietary quality. Consequently, associations between maternal vitamin B_12_ deficiency and outcomes such as preeclampsia, gestational diabetes mellitus, fetal growth restriction, and offspring neurodevelopment should be interpreted as associations rather than evidence of causality. Second, substantial diagnostic heterogeneity exists across studies. Included studies used vitamin B_12_ thresholds ranging from approximately 148 to 350 pg/mL together with different laboratory assays and functional biomarkers, limiting direct comparison between studies and contributing to the wide variation in reported prevalence estimates described in Section 3.1.

Third, randomized evidence remains comparatively limited. The recent Cochrane review included only five randomized controlled trials involving 984 pregnant women and concluded that vitamin B_12_ supplementation may improve maternal and infant vitamin B_12_ status, whereas evidence supporting longer-term clinical outcomes remains insufficient (73). Although the MATCOBIND trial is well conducted and highly relevant to South Asian populations, it remains a single study with relatively short follow-up and an active comparator rather than a placebo control. Independent replication and longer-term follow-up are therefore required before its findings can be generalized.

Finally, as a narrative review, this synthesis is inherently susceptible to selective inclusion and variation in the weighting of evidence. Although the literature search followed a structured and transparent approach, the review protocol was not prospectively registered, and a formal risk-of-bias assessment of individual studies was not undertaken. In addition, the literature search was limited to PubMed/MEDLINE and Scopus and to English-language publications, which may have resulted in the omission of relevant evidence indexed in regional databases or published in local languages. These methodological limitations should be considered when interpreting the conclusions presented in this review. Overall, the available evidence supports targeted screening and treatment of women at high risk of vitamin B_12_ deficiency but remains insufficient to support universal supplementation policies during pregnancy.

### 4.6 Conclusion

Maternal vitamin B_12_ deficiency is highly prevalent in South Asia, is driven by persistent structural determinants, and is associated predominantly through observational evidence—with a broad spectrum of adverse maternal and offspring outcomes linked to disturbed one-carbon metabolism, hyperhomocysteinaemia, and altered methylation. The interaction between low vitamin B_12_ status and high folate exposure, particularly in settings where high-dose folic acid supplementation is routinely provided, further emphasizes the importance of considering vitamin B_12_ status during pregnancy.

Vitamin B_12_ supplementation is safe during pregnancy and lactation and consistently improves biochemical markers of deficiency. However, evidence demonstrating that these biochemical improvements translate into clinically meaningful maternal and offspring benefits remains limited. Although recent randomized trials, including MATCOBIND, provide encouraging findings, the inconsistent translation of biochemical correction into improved clinical outcomes indicates that the current evidence does not support universal vitamin B_12_ supplementation during pregnancy.

A targeted, risk-based approach therefore represents the most evidence-supported and pragmatic strategy for South Asian antenatal care. This approach should prioritize women with established risk factors for deficiency, including vegetarian or vegan diets, limited dietary diversity, socioeconomic disadvantage, suggestive clinical features, or confirmed biochemical deficiency, while maintaining appropriate folic acid supplementation. Women with confirmed deficiency should receive treatment, whereas those with major risk factors should undergo biochemical assessment. Such a strategy would allow vitamin B_12_ deficiency to be identified and treated within existing antenatal-care structures without prematurely committing to universal supplementation in the absence of sufficiently strong clinical outcome evidence.

Future research should prioritize pregnancy-specific diagnostic thresholds, adequately powered randomized trials beginning in the periconceptional period and extending follow-up into childhood, independent replication of recent intervention findings, and cost-effectiveness evaluations of risk-based screening and targeted supplementation in South Asian populations.
