# Supplementary material for: Maternal vitamin B12 deficiency in pregnancy: burden, associated outcomes, and implications for antenatal care in South Asia—a narrative review
Source: Front Nutr. 2026 Oct 22;13:1896162. doi: 10.3389/fnut.2026.1896162 (PMC13736174; doi:10.3389/fnut.2026.1896162)
Supplement: Supplementary file 1 [file Supplementary_file_1.docx]

**Supplementary Table S1. Database-specific search strategies, search dates, and records retrieved**

| Database | Search strategy and limits | Date searched | Records retrieved (n) |
| --- | --- | --- | --- |
| PubMed/MEDLINE | Medical Subject Headings (MeSH) were combined with free-text terms searched within titles and abstracts using the [tiab] field tag. Searches included terms relating to vitamin B₁₂ and one-carbon metabolism, pregnancy and maternal health, and maternal, fetal, neonatal, and offspring outcomes. Limits applied were: publication date 2000/01/01–2026/03/31, English language, and human studies. The complete PubMed search strategy is provided below. | 20 April 2026 | 2,115 |
| Scopus | Searches were performed using the TITLE-ABS-KEY field with Boolean operators and truncation where appropriate. Limits applied were: PUBYEAR >1999 AND PUBYEAR <2027, LANGUAGE (English), DOCTYPE (Article OR Review), and relevant subject areas (Medicine, Biochemistry, Nursing, and Health Sciences). The complete Scopus search strategy is provided below. | 20 April 2026 | 5,218 |
